# Synaptic dysfunction and glial activation markers throughout aging and early neurodegeneration: a longitudinal CSF biomarker-based study

**DOI:** 10.1186/s13024-025-00901-5

**Published:** 2025-10-17

**Authors:** Mariana I. Muñoz-García, Yuetiva Deming, Ferran Lugo-Hernández, Sterling Johnson, Sanjay Asthana, Gwendlyn Kollmorgen, Clara Quijano-Rubio, Cynthia Carlsson, Ozioma C. Okonkwo, David Pérez-Martinez, Alberto Villarejo-Galende, Kaj Blennow, Marc Suárez-Calvet, Henrik Zetterberg, Barbara B. Bendlin, Estrella Morenas-Rodríguez

**Affiliations:** 1 Memory Unit, Neurology Department, Hospital Universitario 12 de Octubre, Madrid, España; 2 Neurodegenerative Diseases Research Group, Instituto de Investigación Sanitaria Hospital 12 de Octubre (imas12), Madrid, Spain; 3 Wisconsin Alzheimer’s Disease Research Center, University of Wisconsin School of Medicine and Public Health, University of Wisconsin-Madison, Madison, WI USA; 4 Barcelonaβeta Brain Research Center (BBRC), Pasqual Maragall Foundation, Wellington 30, Barcelona, 08005 Spain; 5 Hospital del Mar Research Institute, C. del Dr. Aiguader 88, Barcelona, 08003 Spain; 6 Roche Diagnostics GmbH, Penzberg, Germany; 7 Roche Diagnostics International Ltd, Rotkreuz, Switzerland; 8 Department of Psychiatry and Neurochemistry, Institute of Neuroscience and Physiology, The Sahlgrenska Academy at the University of Gothenburg, Mölndal, Sweden; 9 Servei de Neurologia, Hospital del Mar, Passeig Marítim 25-29, Barcelona, 08003 Spain; 10 Clinical Neurochemistry Laboratory, Sahlgrenska University Hospital, Mölndal, Sweden; 11 Department of Neurodegenerative Disease, UCL Institute of Neurology, Queen Square, London, UK; 12 UK Dementia Research Institute at UCL, London, UK; 13 Hong Kong Center for Neurodegenerative Diseases, InnoHK, Hong Kong, China; 14 Centre for Brain Research, Indian Institute of Science, Bangalore, India

**Keywords:** Microglia, Synaptic function, Aging, Neurodegeneration, TREM2

## Abstract

**Background:**

Synaptic homeostasis, maintained by microglia and astroglia, is disrupted throughout aging and early on in neurodegenerative diseases. Our aim was to study the relationship between TREM2-dependent microglial reactivity, astroglial response and synaptic dysfunction in two longitudinal cohorts of cognitively healthy volunteers and determine whether this relationship is influenced by AD core biomarkers.

**Methods:**

We analyzed cross-sectional and longitudinal associations between cerebrospinal fluid levels of soluble TREM2 (sTREM2), astroglial markers (GFAP, S100B), and synaptic markers (neurogranin, α-synuclein) in cognitively unimpaired participants from the Wisconsin Registry for Alzheimer’s Prevention (WRAP) and the Alzheimer’s and Families (ALFA+) cohort. Biomarkers were quantified using validated immunoassays (NeuroToolKit, Roche), with sTREM2 measured using an in-house MSD-based assay in the WRAP cohort. Linear regression and linear mixed-effects models were used, both unadjusted and adjusted for Aβ42 and p-tau. Subgroup analyses were performed based on AT classification, *APOE*-ε4 status, and median splits of Aβ42/Aβ40 ratio and p-tau, to capture profiles suggestive of early AD-related neuropathogenesis.

**Results:**

We found significant cross-sectional associations between sTREM2 and α-synuclein, as well as between sTREM2 and S100B, in subgroups exhibiting AD-related biomarker profiles. Longitudinally, lower baseline neurogranin and α-synuclein and higher S100B predicted greater increases in sTREM2 over time independently of AD-related markers in the WRAP cohort (β = −0.02, *p* = 0.006; β = −0.02, *p* = 0.01; β = 0.02, *p* = 0.03, respectively). In ALFA+, lower baseline α-synuclein also predicted a greater subsequent longitudinal increase in sTREM2, but only among individuals with Aβ42/Aβ40 ratio above the median (β = -0.01, *p* = 0.05). Notably, higher baseline sTREM2 was associated with a smaller longitudinal increase in neurogranin in both cohorts (β = -0.01, *p* = 0.03 for WRAP, β = -0.01, *p* = 0.04 in ALFA+).

**Conclusions:**

Synaptic dysfunction markers at baseline influence the longitudinal dynamics of CSF sTREM2 independently of AD-pathology related biomarkers throughout aging and earliest stages of neurodegeneration. In turn, higher baseline sTREM2 is associated with more stable neurogranin levels over time. These results suggest an independent interaction between synaptic dysfunction and TREM2-dependent microglial activation throughout aging and early neurodegeneration beyond AD pathology.

**Supplementary Information:**

The online version contains supplementary material available at 10.1186/s13024-025-00901-5.

## Background

Synaptic dysfunction and glial reactivity play crucial roles in the early stages and progression of neurodegenerative diseases, as well as throughout the aging process [1, 2]. Experimental evidence suggests that microglia and astrocytes play critical roles in regulating synaptic plasticity during both physiological aging and disease [3–5]. Microglial-derived proteins such as TGF-β1 and C1q have been identified as crucial signals that impact synaptic protein homeostasis and influence synaptic maintenance in the aging brain [6, 7]. Astrocytes contribute through neurotransmitter clearance, ion balance regulation, and gliotransmitter release, which affect short- and long-term synaptic plasticity [3, 8, 9]. Thus, both glial cell types are well-positioned to sense early disruptions in synaptic activity and potentially interact with synaptic failure throughout both aging and disease [3].

Microglial and astroglial responses to early protein aggregation have also been identified as key processes in neurodegenerative diseases [10, 11]. Single-cell sequencing technologies have identified dynamic microglial populations transitioning from homeostatic to disease-associated profiles in response to initial protein aggregation [12]. Loss-of-function genetic variants affecting the microglial protein triggering receptor expressed on myeloid cells 2 (TREM2) increase AD risk by preventing microglia from transitioning to a protective disease-associated state [13].

Similarly, disease-associated astrocytes are often found surrounding pathological protein aggregates in various neurodegenerative diseases and appear to be induced by activated microglia through pro-inflammatory cytokines [14]. This activation leads to a loss of astrocytic homeostatic roles, including maintaining synaptic functionality, as shown in animal-based studies [15]. These findings suggest active synapse regulation by microglia and astroglia in neurodegeneration [16]. Nonetheless, the association between glial responses and synaptic dysfunction remains largely unexplored in human cohorts in the framework of aging and neurodegeneration.

Synaptic function can be assessed in live human cohorts by measuring synapse-related biomarkers in cerebrospinal fluid (CSF). Neurogranin is a postsynaptic protein widely studied in CSF. Reduced CSF levels of neurogranin have been described in Parkinson’s disease (PD), dementia with Lewy bodies (DLB), and preclinical Alzheimer’s disease (AD) [17–19]. However, most studies in AD report elevated CSF neurogranin across the continuum of disease, as well as correlation with CSF p-tau, likely reflecting progressive dendritic pathology and/or neuronal hyperactivity [20–23]. CSF alpha-synuclein (α-syn), a crucial presynaptic protein, has different changes in concentration depending on the disease context, stage, and analytical method [24–26]. Discrepancies in CSF α-syn levels highlight the multifactorial nature of α-syn biology, which encompasses synaptic dysfunction, aggregation, neurodegeneration, and axonal remodeling [18, 27–29]. Thus, these synaptic proteins are probably appropriate synapse-related markers during healthy aging or early stages of neurodegenerative processes but should be interpreted carefully in the context of advanced neurodegeneration.

Glial function can also be studied in humans through biomarkers in biofluids, offering a translational bridge between experimental findings and clinical observations. The soluble cleavage product of the microglial protein TREM2 (sTREM2) serves as a marker of the cell-autonomous microglial activation response [28, 29]. Longitudinal biomarker-based data from autosomal-dominant AD suggests that the main trigger of TREM2-dependent microglial response is initial Aβ aggregation, decades before the first symptoms appear [11]. Higher baseline levels and longitudinal increase of sTREM2 in CSF are associated with slower cognitive decline and reduced amyloid accumulation in AD patients, which supports the protective role of microglia [11, 30].

Additionally, astroglial reactivity marker GFAP has been proposed to mediate the transition between soluble and insoluble Aβ and represent a possible connection between amyloidosis and tau-related neurodegeneration in sporadic AD [10, 31]. Another important astroglial activation marker measurable in CSF is S100 calcium-binding protein B (S100B). This marker is proposed to play a role in modulating synaptic plasticity and microglial response [32, 33]. These biomarkers are useful for monitoring the dynamic roles of microglia and astroglia in clinical cohorts throughout aging and disease stages.

Understanding the interplay between synaptic dysfunction and glial responses is essential for characterizing the biological underpinnings of cognitive decline in aging and neurodegenerative diseases. These processes reflect key mechanisms underlying cognitive impairment and represent some of the earliest events driving disease progression, potentially opening new windows for therapeutic intervention. Nonetheless, the temporal relationships among presynaptic (α-syn), postsynaptic (neurogranin), microglial (sTREM2), and astroglial (GFAP, S100B) markers remain poorly understood, particularly in asymptomatic individuals, as very few studies directly investigated this association.

In this study, we leverage longitudinal CSF biomarker data from two independent cohorts of cognitively unimpaired, late-middle-aged individuals to investigate how synaptic and glial signals crosstalk with TREM2-dependent microglial activation. By integrating cross-sectional and longitudinal analyses across populations with differing AD risk profiles, we aim to uncover early synapse-to-glia signaling dynamics that distinguish physiological aging from the preclinical phase of neurodegeneration—ultimately informing the development of precision strategies for early intervention.

## Materials and methods

### Patient cohorts

We conducted a longitudinal observational study using CSF biomarker data from two independent cohorts of cognitively unimpaired adults, aiming to assess associations between synaptic and glial biomarkers and microglial activation over time. The first cohort of participants stemmed from the Wisconsin Registry for Alzheimer’s Prevention (WRAP) study; an observational cohort first established in 2001. The WRAP study initially enrolled participants at midlife with a mean age of 54 and parental history of probable AD dementia and thus, at risk for late onset dementia. Since 2004, it also included participants without parental history of dementia to better understand its role in the risk of dementia. Genetic and clinical data was gathered initially, and subjects were followed longitudinally with neuropsychological evaluation, self-reported medical and lifestyle data, laboratory tests, and optional lumbar puncture (LP) at different time points (approximately every two years). Further details about the cohort are discussed elsewhere [34]. This study uses a subset of 239 WRAP participants, with available CSF samples, 116 of them with available longitudinal data, out of the 1561 total participants [34].

We repeated analyses in a confirmation cohort, the ALFA + cohort, a longitudinal observational cohort nested within the ALFA (for ALzheimer and FAmilies) parent cohort. The ALFA study was established between 2013 and 2014 and recruited 2,743 cognitively unimpaired individuals, primarily first-degree descendants of patients with sporadic AD, aged 45 to 75 years. Participants were extensively characterized at baseline, including sociodemographic, clinical, lifestyle, and cognitive measures, with additional data collected on modifiable risk factors and *APOE* genotype. Within the ALFA cohort, a subset was selected for the ALFA + study based on risk profile (*APOE* and family history status), and 400 of those participants were available for cross-sectional analyses. ALFA + involves more detailed longitudinal phenotyping, including fluid biomarker collection. Baseline visits occurred between 2016 and 2019, with follow-up assessments every three years. In the present study, at baseline, 15 blood-based biomarkers were analyzed, and during the second wave (V2, begun in 2019), CSF biomarkers such as Aβ42, p-tau, t-tau, GFAP, neurogranin, S100, α-syn, sTREM2, and others were measured [35]. A subset of 259 had available longitudinal data at the time of analyses.

### CSF biomarker quantification

Synapse-related biomarkers including neurogranin and α-syn, and astroglial markers S100B and GFAP, as well as Aβ40 were measured using the NeuroToolKit (NTK) in both the WRAP and ALFA + cohorts, as a panel of exploratory prototype assays [25, 36, 37]. The specific cleaved sTREM2 isoform was measured in the CSF of participants included in the WRAP cohort by an in-house immunoassay in the MSD platform as previously reported [11, 37]. Total sTREM2 was quantified in the ALFA + cohort by the NTK [36]. AD core biomarkers including Aβ42, total tau (t-tau) and tau phosphorylated at threonine 181 (p-tau) in CSF were quantified by the commercially available Elecsys^®^ immunoassays, as described elsewhere [36]. Biomarkers Aβ42, Aβ40, pTau, t-tau and S100B were measured using the Cobas^®^ e 601 analyzer, and the remaining on a Cobas^®^ e 411 analyzer (both Roche Diagnostics International Ltd) [36]. All NTK measurements for both cohorts were performed in singlicate at the Clinical Neurochemistry Laboratory at the University of Gothenburg (Sweden). In the WRAP cohort, we defined positivity in the AT classification as A+ for Aβ42/Aβ40 ratio < 0.046 and T+ for p-tau >24.8 pg/mL; in the ALFA + cohort, as Aβ42/Aβ40 ratio < 0.071 and p-tau >24 pg/mL. 

### Statistical analysis

Only participants with complete data (all measurements for biomarkers and covariate data for each described model) were included in the analyses; no imputation was performed. We first tested for normality of the distribution for each biomarker using the Shapiro test. CSF Aβ42, Aβ42/Aβ40 ratio, p-tau, t-tau, sTREM2, neurogranin, α-syn, S100B, and GFAP did not follow a normal distribution and were thus log10-transformed. To describe the data, we stratified participants according to *APOE* status, medians of Aβ42/Aβ40 ratio and p-tau, and we used χ2 tests for categorical variables, and t-student or ANOVA for continuous variables to compare groups. We stratified the cohort according to medians of Aβ42/Aβ40 ratio and p-tau to better approach the contribution of the earliest AD-related pathological changes, since this cohort was composed of healthy participants with a low percentage of amyloid and p-tau positivity. This methodology is supported by findings showing differential longitudinal cognitive profiles based on preclinical AD-biomarker changes, when dividing the Aβ42 and p-tau values according to cohort-specific tertiles and medians [38].

For cross-sectional analysis, we calculated partial correlations adjusted by age across all biomarkers with the Pearson method. We then performed linear regression analysis to study the association between sTREM2 and synaptic function biomarkers, using TREM2 both as the main independent variable and the dependent outcome variable. For each analysis, we use two linear regression models: Model 1, adjusted for age and gender, and Model 2, further adjusted for baseline Aβ42 and p-tau levels. Analyses were conducted in the entire cohort and stratified by subgroups based on the median Aβ42/Aβ40 ratio, p-tau levels, Aβ or p-tau marker positivity (AT classification), and *APOE* carriage status, to better capture different preclinical stages of the AD continuum. Furthermore, we repeated the models including interaction terms between the independent biomarker and Aβ42 and p-tau, as continuous variables. The overall aim was to evaluate whether these associations were influenced by an underlying initial AD pathological process.

For longitudinal analyses, we performed linear mixed effects models with random intercepts to account for within-subject variability over time, based on longitudinal data from 116 participants in the WRAP cohort and 259 in the ALFA + cohort. The WRAP cohort participants had mainly one follow-up visit (20 had 3 and 6 had 4 visits), while all participants in the ALFA + cohort had only one follow-up visit. For this reason, the statistical model included only random intercepts (not random slopes) to avoid convergence issues, which allowed to model between-subject variability without overfitting. Linear mixed-effects models were implemented using the lme4 package. Univariate models were used to assess the effect of baseline biomarkers (predictor) on the longitudinal change of the outcome biomarker. We included an interaction term between the baseline biomarker and time to understand how the relationship between biomarkers changed over time. The time variable was modeled as a continuous variable centered at baseline (time of first LP).

We used sTREM2 both as the outcome biomarker and as the predictor for each synaptic biomarker. We adjusted the models by age, gender, and continuous values of Aβ42, and p-tau. We also calculated the interaction between time and baseline biomarker levels divided by medians. We performed sensitivity analysis by performing analysis in previously described subgroups. Given the exploratory nature of the study, p-values were considered nominally significant at *p* < 0.05 without correction for multiple comparisons. All statistical analyses were performed with R software (http://www.r-project.org/) and RStudio (last updated version 2024.12.0 + 467).

## Results

### WRAP cohort

Demographic and baseline biomarker characteristics of WRAP participants are summarized in Table 1. Participants with both an Aβ42/Aβ40 ratio below the median and p-tau levels at or above the median were older at the time of LP and had a higher frequency of *APOE* ε4 carriers, compared to other subgroups. No differences were observed in gender, parental history of dementia, ethnicity, or MMSE score. As expected, groups defined by the median of Aβ42/Aβ40 ratio differed in percentage of amyloid positivity according to Aβ42/Aβ40 ratio and p-tau/Aβ42 ratio, as well as tau positivity according to pre-established cut-offs. Groups defined by median p-tau, also differed in percentage of amyloid positivity according to Aβ42/Aβ40 ratio and p-tau/Aβ42 ratio.

The biomarker profiles adjusted by age are also shown in Table 1. The group of participants with an Aβ42/Aβ40 ratio below the median showed biomarkers congruent with first stages of amyloid aggregation: lower Aβ42, higher t-tau and p-tau, lower Aβ42/Aβ40 ratio than participants with an Aβ42/Aβ40 ratio above the median. When stratifying by p-tau median, participants with p-tau levels above the median also had a biomarker profile suggestive of first stages within the AD continuum, except for having higher baseline Aβ42, but this was compensated with a significantly lower Aβ42/Aβ40 ratio. Cross-sectionally, participants with p-tau levels above the median had higher levels of sTREM2, GFAP, S100B, neurogranin and ⍺-syn than participants with p-tau levels below the median (Table 1). In contrast, levels of the studied proteins did not significantly differ between groups stratified according to Aβ42/Aβ40 ratio. Partial correlations adjusted by age between studied markers in the entire cohort are summarized in Supplementary Fig. 1.

**Table 1:** Demographics and baseline biomarkers in the WRAP (Wisconsin’s registry for alzheimer Prevention) cohort

	Overall	Aβ42/Aβ40 ratio ≥ median (2)	Aβ42/Aβ40 ratio < median	*p*	*p*-tau ≥ median (4)	*p*-tau < median	*p*
n	239	120	119		120	119	
Age at time of LP, y [mean (SD)]	61.4 (7.2)	60.1 (7.47)	62.8 (6.6)	***0.003***	62.5 (7.30)	60.4 (6.94)	***0.021***
Gener, Male [n (%)]	89 (37.2)	45 (37.5)	44 (37.0)	*1.000*	39 (32.5)	50 (42.0)	*0.165*
PH of dementia, Yes [n (%)]	177 (74.1)	91 (75.8)	86 (72.3)	*0.631*	89 (74.2)	88 (73.9)	*1.000*
Ethnicity [n (%)]				*0.454*			*0.556*
White	230 (96.2)	115 (95.8)	115 (96.6)		116 (96.7)	114 (95.8)	
Other (1)	9 (3.8)	5 (4.2)	4 (3.4)		4 (3.3)	4 (4.1)	
ApoE 𝛆4, Non-carrier [n (%)]	151 (63.2)	91 (75.8)	60 (50.4)	***< 0.001***	67 (55.8)	84 (70.6)	***0.026***
MMSE [mean (SD)]	29.3 (0.9)	29.3 (0.89)	29.3 (0.94)	*0.518*	29.29 (1.00)	29.3 (0.82)	*0.902*
Education, y [mean (SD)]	16.3 (2.4)	16.1 (2.45)	16.4 (2.24)	*0.213*	16.27 (2.35)	16.2 (2.36)	*0.896*
Amyloid + [n (%)] (3)	46 (19.2)	0 (0.0)	46 (38.7)	***< 0.001***	41 (34.2)	5 (4.2)	***< 0.001***
Tau + [n (%)] (5)	25 (10.5)	4 (3.3)	21 (17.6)	***0.001***	25 (20.8)	0 (0.0)	***< 0.001***
Positive ptau/ab42 ratio [n (%)]	35 (14.6)	0 (0.0)	35 (29.4)	***< 0.001***	31 (25.8)	4 (3.4)	***< 0.001***
				*p (adj by age)*			*p (adj by age)*
Aβ42, pg/mL [mean (SD)]	881 (375)	1097 (337)	663 (272)	***< 0.001***	1009 (419)	751 (269)	***< 0.001***
Aβ40, pg/mL [mean (SD)]	14,024 (4395)	14,494 (4072)	13,550 (4667)	***0.027***	16,995 (3570)	11,028 (2841)	***< 0.001***
Aβ42/Aβ40 ratio [mean (SD)]	0.06 (0.02)	0.08 (0.01)	0.05 (0.01)	***< 0.001***	0.06 (0.02)	0.07 (0.01)	***< 0.001***
T- tau, pg/mL [mean (SD)]	196 (63)	183.3 (52.8)	208 (77.6)	***0.0374***	246 (56.6)	145 (25.9)	***< 0.001***
P-tau, pg/mL [mean (SD)]	17.3 (6.75)	15.9 (4.93)	18.6 (7.99)	***0.0224***	22.2 (6.12)	12.3 (2.22)	***< 0.001***
sTREM2 MSD, ng/mL [mean (SD)]	7.63 (2.88)	7.57 (2.78)	7.69 (2.99)	*0.539*	8.69 (2.95)	6.56 (2.37)	***< 0.001***
sTREM2 NTK, ng/mL [mean (SD)]	7.78 (2.29)	7.82 (2.18)	7.73 (2.41)	*0.152*	8.92 (2.34)	6.62 (1.54)	***< 0.001***
Neurogranin, pg/mL [mean (SD)]	771 (298)	741 (248)	801 (339)	*0.263*	986 (255)	554 (138)	***< 0.001***
𝛂- synuclein, pg/mL [mean (SD)]	152 (64.2)	149 (56.4)	155 (71.2)	*0.860*	193 (61.9)	110 (30.9)	***< 0.001***
S100B, pg/mL [mean (SD)]	1.16 (0.30)	1.15 (0.29)	1.17 (0.30)	*0.949*	1.20 (0.28)	1.12 (0.30)	*0.065*
GFAP, ng/mL [mean (SD)]	8.69 (3.03)	8.67 (3.22)	8.72 (2.84)	*0.253*	9.72 (3.14)	7.66 (2.52)	***< 0.001***

(1) Other: American Indian or Alaska Native, Asian, Black or African American and other (2) Aβ42/Aβ40 median = 0.067 (3) The cut-off for amyloid (A) positivity according to the Aβ42/Aβ40 ratio is 0.046. (4) P-tau median = 15.94 pg/mL. (5) The cut-off for tau (T) positivity according to P-tau is 24.8 pg/mL. LP: lumbar puncture. PH: Parental history. MMSE: mini-mental state examination score

**Fig. 1:**
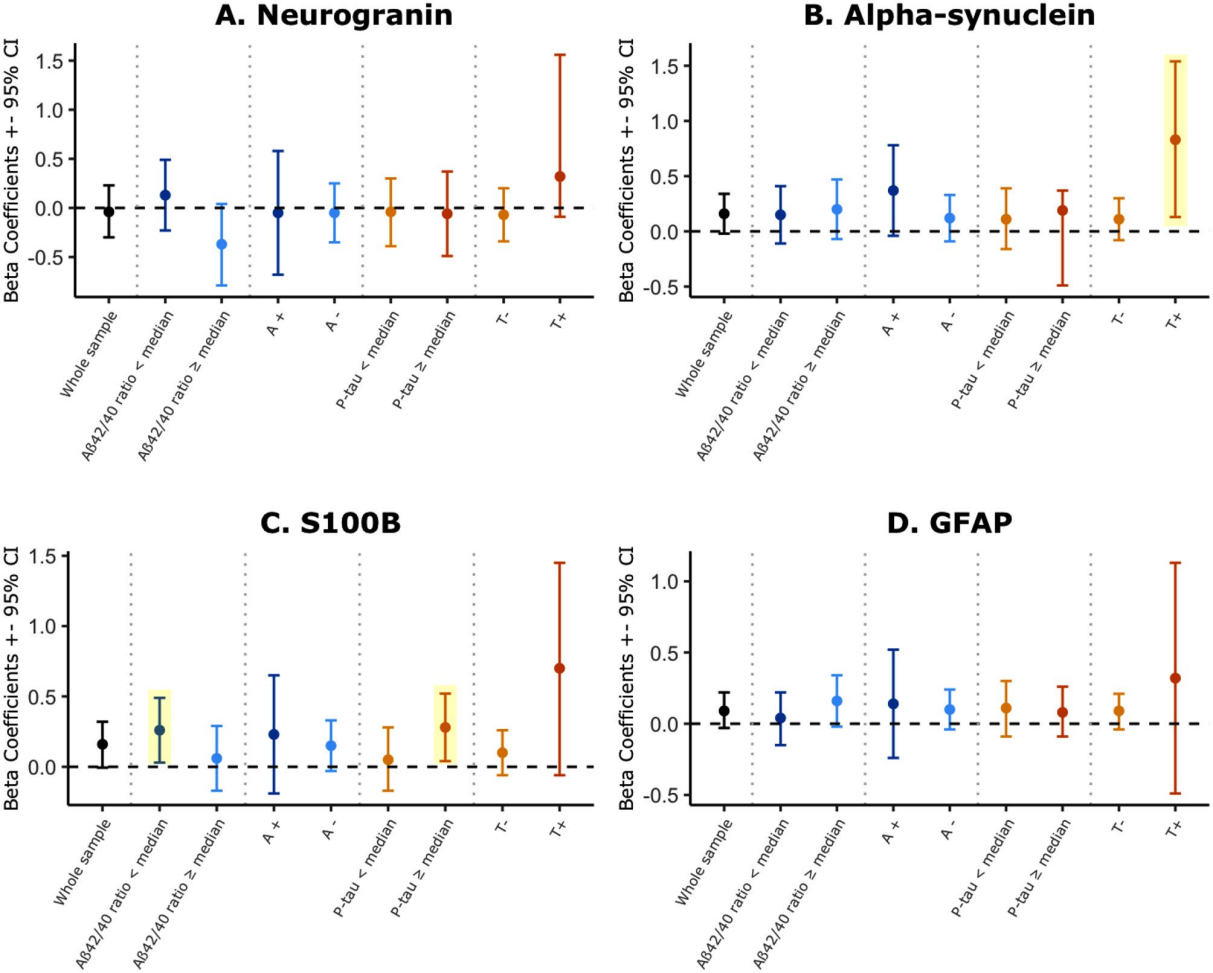
Cross-sectional associations between biomarkers and sTREM2. The figure represents the Beta-coefficient, extracted from linear regression models, for each biomarker (panel **A**: Neurogranin, panel **B**: Alpha-synuclein, panel **C**: s100b, and panel **D**: GFAP) as predictor of sTREM2 levels. All models are adjusted for age, sex, Aβ42, and p-tau. Results are shown in the whole sample and stratified by subgroups according to medians (Aβ42/Aβ40 median = 0.067; p-tau median = 15.94 pg/mL), and cut-offs for amyloid (A+) positivity (Aβ42/Aβ40 < 0.046) and tau (T+) positivity (p-tau > 24.8 pg/mL). Values shadowed in yellow represent significant associations after adjusting for covariates. Error bars represent 95% confidence intervals

In cross-sectional analyses, we performed linear regression models adjusted for age and gender (Model 1), and then further adjusted for baseline Aβ42 and p-tau levels (Model 2), as well as after stratification by subgroups based on the median Aβ42/Aβ40 ratio, p-tau levels, Aβ or p-tau marker positivity (A/T classification), and *APOE* carriage status. These are summarized in Supplementary Table 1. We found significant cross-sectional associations between sTREM2 and GFAP (β = 0.26, *p* = 0.0001), S100B (β = 0.28, *p* = 0.002), neurogranin (β = 0.37, *p* < 0.0001), and ⍺-syn (β = 0.37, *p* < 0.0001), in the whole cohort using Model 1 (Supplementary Table 1). In contrast, after adjusting for Aβ42 and p-tau baseline levels (Model 2), we only found a trend for an association in the whole sample between sTREM2 and S100B (β = 0.16, *p* = 0.06), and between sTREM2 and ⍺-syn (β = 0.16, *p* = 0.09), indicating an influence of AD related markers on the previous associations. The results for Model 2 are shown in Fig. 1. In subgroups stratified by AD biomarker profiles, we found a significant cross-sectional association between sTREM2 and ⍺-syn CSF levels in T + participants even after AD-related markers adjustment, indicating that the cross-sectional relationship is not influenced by AD-related biomarkers (β = 0.83, *p* = 0.02) shown in Fig. 1B and Supplementary Table 1.

Additionally, we found a significant association between sTREM2 and S100B in participants with Aβ42/Aβ40 ratio below the median (β = 0.26, *p* = 0.03) and in participants with p-tau levels above the median (β = 0.28, *p* = 0.01) (Fig. 1C and Supplementary Table 1) after AD-related marker adjustment. This suggests that the association between sTREM2 and S100B is present in individuals with a biomarker profile indicative of first stages of an AD pathology and is not mediated by Aβ42 or p-tau levels. We did not find any other significant cross-sectional association between sTREM2 and neurogranin or GFAP in models adjusted by Aβ42 and p-tau. The cross-sectional associations between sTREM2 and studied biomarkers were not affected by the *APOE* 𝛆4 allele carriage status, whether included as a covariate in regression models or assessed in stratified analyses (Supplementary Table 1). Furthermore, interaction terms were tested using continuous values of Aβ42 and p-tau; none were statistically significant and are not shown.

Then, we examined whether baseline astroglial response and synapse-related markers influenced the longitudinal dynamics of sTREM2 using linear mixed models. Two models were applied: Model 1, adjusted for age and gender, and Model 2, further adjusted for baseline Aβ42 and p-tau levels. Among the 116 participants, follow-up data were available for 90 individuals with one visit, 20 with two visits, and 6 with three visits. The adjustment for baseline Aβ42 and p-tau did not alter the association between baseline astroglial and synaptic markers and the longitudinal change in sTREM2. Nevertheless, to evaluate the independent associations between astroglial and synaptic markers and the longitudinal change in sTREM2, we focused on the adjusted models (Model 2). A summary of these models is provided in Supplementary Table 2.

**Fig. 2:**
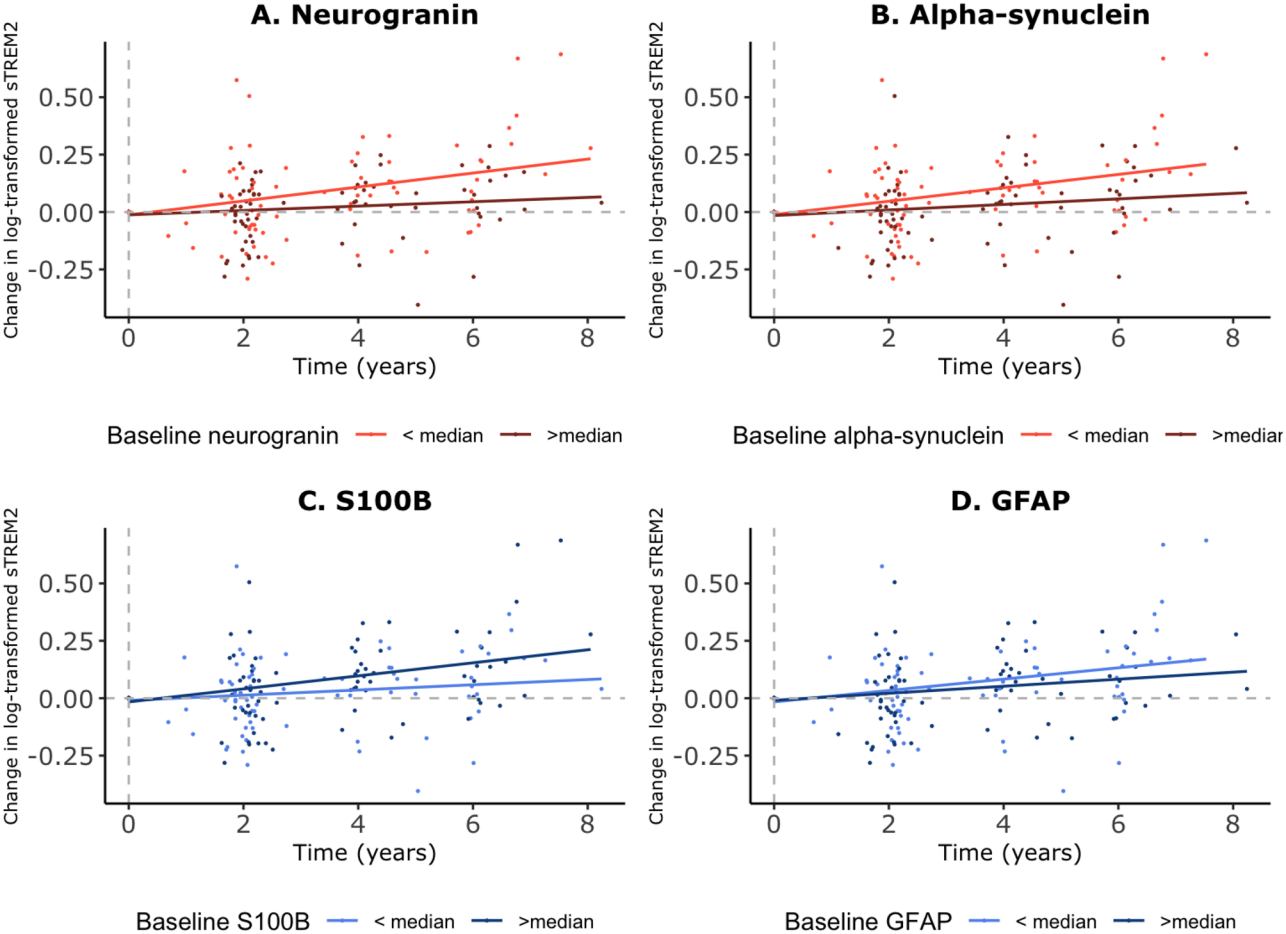
Longitudinal associations between baseline synaptic and astroglial biomarkers and sTREM2. *Figures show the change from baseline levels in log-transformed values across time for sTREM2*,* according to baseline levels* of each synaptic or astroglial biomarker. The dotted line represents the intercept (set to 0). Linear mixed models that represent these plotted associations are adjusted by age, gender, Aβ42 and p-tau. (**A**) *Neurogranin*: β-coefficient for interaction with time = -0.04, *p* = 0.0001 (continuous values), β-coefficient for interaction with time = -0.02, *p* = 0.006 (comparing > median vs. < median). (**B**) *Alpha-synuclein*: β-coefficient for interaction with time = -0.03, *p* = 0.002 (continuous values), β-coefficient for interaction with time = -0.02, *p* = 0.01 (comparing > median vs. < median). (**C**) *S100B*: β-coefficient for interaction with time = 0.02, *p* = 0.32 (continuous values), β-coefficient for interaction with time = 0.02, *p* = 0.03 (comparing > median vs. < median). (**D**) *GFAP*: β-coefficient for interaction with time = -0.004, *p* = 0.73 (continuous values). β-coefficient for interaction with time = -0.01, *p* = 0.12 (comparing > median vs. < median)

Concerning the synapse-related markers, lower levels of baseline neurogranin (β-coefficient for interaction with time = -0.04, *p* = 0.0002) and ⍺-synuclein (β-coefficient for interaction with time = -0.03, *p* = 0.004) significantly predicted a larger subsequent longitudinal increase in sTREM2 CSF levels over time. When stratifying baseline neurogranin and ⍺-synuclein by their median values, we consistently observed that levels below the median were significantly associated with an increase in sTREM2 over time (Fig. 2A and B). Regarding the astroglial markers GFAP and S100B, we only observed a significant association with baseline S100B when stratifying by its median, while no significant associations were found between baseline GFAP and the longitudinal change of sTREM2 levels. Participants with baseline S100B levels above the median showed a significantly greater subsequent longitudinal increase in CSF sTREM2 levels (β-coefficient for the interaction between time and S100B above median = 0.02, *p* = 0.03), as shown in Fig. 2C and Supplementary Table 2. These longitudinal associations remained significant when stratifying by Aβ42/Aβ40 ratio medians, p-tau medians, amyloid or p-tau positivity cut-offs, or *APOE* 𝜀4 carrier status (Supplementary Table 2).

**Fig. 3:**
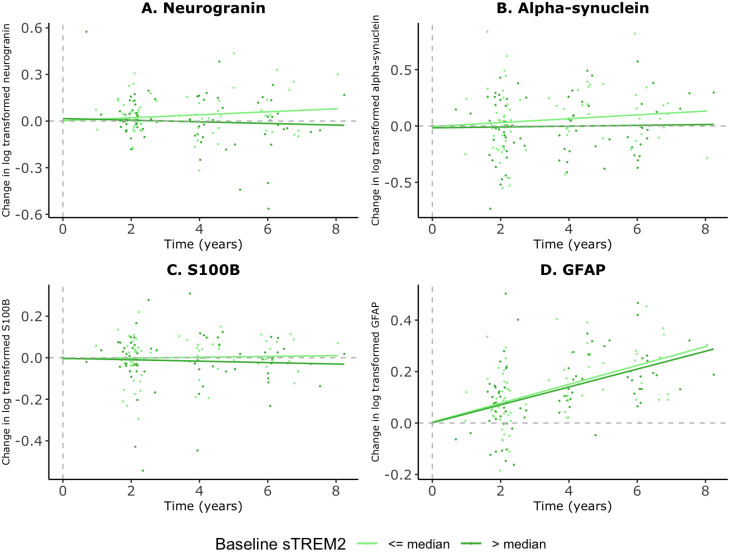
Longitudinal associations between baseline sTREM2 and synaptic and astroglial biomarkers. Figures show the change from baseline levels in log-transformed values across time for each biomarker, according to baseline sTREM2 levels. The dotted line represents the intercept (set to 0). Linear mixed models that represent these plotted associations are adjusted by age, gender, Aβ42 and p-tau. (**A**) Neurogranin: β-coefficient for interaction with time = -0.03, p = 0.0001 (continuous values). β-coefficient for interaction with time = -0.01, p = 0.03 (comparing > median vs < median). (**B**) ⍺-syn: β-coefficient for interaction with time = -0.03, p = 0.08 (continuous values). β-coefficient for interaction with time = -0.01, p = 0.33 (comparing > median vs < median). (**C**) S100B: β-coefficient for interaction with time = -0.006, p = 0.36 (continuous values). -coefficient for interaction with time = -0.003, p = 0.55 (comparing > median vs < median). (**D**) GFAP: β-coefficient for interaction with time = -0.006, p = 0.40 (continuous values). β-coefficient for interaction with time = -0.004, p = 0.46 (comparing > median vs < median)

Finally, we evaluated whether baseline sTREM2 levels influenced the subsequent longitudinal changes in synapse-related markers and astroglial response. After adjusting for Aβ42 and p-tau, we found that higher baseline sTREM2 levels were significantly associated with a diminished longitudinal increase in neurogranin over time (β-coefficient for interaction with time =-0.03, *p* = 0.0001) (Fig. 3A). No other significant associations were observed between baseline sTREM2 levels and the longitudinal changes of ⍺-synuclein, S100B or, GFAP (Fig. 3B, C and D).

### ALFA + cohort

To replicate our findings in an independent sample, we applied the same analytic pipeline to the ALFA + cohort. Demographics of this cohort are shown in Table 2. Demographically, the main differences between the cohorts were the proportion of *APOE* 𝛆4 carriers (54% in ALFA + vs. 37% WRAP), which influenced the proportion of A + participants (33.8% vs. 19%), but not T+ (11.9% vs. 10.5%). Subgroup analysis revealed that participants with an Aβ42/Aβ40 ratio below the median and p-tau above the median had profiles closer to the AD continuum. We repeated the same cross-sectional and longitudinal analyses used in the WRAP cohort. Partial correlation results are summarized in Supplementary Fig. 1, showing a similar profile to correlations in the WRAP cohort.

**Table 2:** Demographics and baseline biomarkers for the Alzheimer and Families (ALFA+) cohort

	Overall	Aβ42/Aβ40 ratio ≥ median (2)	Aβ42/Aβ40 ratio < median	*p*	*p*-tau ≥ median (4)	*p*-tau < median	*p*
n	400	200	200		177	176	
Age at time of LP, y [mean (SD)]	61.2 (4.69)	60.4 (4.34)	61.9 (4.90)	***0.001***	61.7 (4.37)	59.9 (4.82)	***< 0.001***
Gener, Male [n (%)]	245 (62.5)	123 (62.8)	122 (62.2)	*1.000*	114 (65.5)	110 (64.3)	*0.906*
Ethnicity [n (%)]				*0.343*			*0.716*
White	394 (98.5)	197 (98.5)	197 (98.5)		175 (98.9)	172 (97.7)	
Other (1)	6 (1.5)	3 (1.5)	3 (1.5)		2 (1.1)	4 (2.3)	
ApoE 𝛆4, Non-carrier [n (%)]	184 (46.0)	128 (64.0)	56 (28.0)	***< 0.001***	80 (45.2)	84 (47.7)	*0.712*
MMSE [mean (SD)]	29.1 (0.99)	29.2 (0.92)	29.1 (1.05)	*0.206*	29.1 (1.10)	29.2 (0.88)	*0.450*
Education, y [mean (SD)]	4.49 (0.91)	4.55 (0.88)	4.42 (0.94)	*0.170*	4.43 (0.97)	4.51 (0.88)	*0.406*
Amyloid + [n (%)] (3)	135 (33.8)	0 (0.0)	135 (67.5)	***< 0.001***	73 (41.2)	43 (24.4)	***0.001***
Tau + [n (%)] (5)	42 (11.9)	10 (5.4)	32 (19.0)	***< 0.001***	42 (23.7)	0 (0.0)	***< 0.001***
Positive ptau/ab42 ratio [n (%)]	50 (14.2)	0 (0.0)	50 (29.8)	***< 0.001***	44 (24.9)	6 (3.4)	***< 0.001***
				*p (adj by age)*			*p (adj by age)*
Aβ42, pg/mL [mean (SD)]	1322 (597)	1661 (601)	983 (350)	***< 0.001***	1607 (713)	1143 (341)	***< 0.001***
Aβ40, pg/mL [mean (SD)]	17,403 (4997)	17,971 (5120)	16,834 (4817)	***0.023***	21,191 (4207)	14,521 (2874)	***< 0.001***
Aβ42/Aβ40 ratio [mean (SD)]	0.08 (0.02)	0.09 (0.01)	0.06 (0.02)	***< 0.001***	0.07 (0.02)	0.08 (0.01)	*0.059*
T- tau, pg/mL [mean (SD)]	201 (72.9)	192 (56.5)	212 (86)	***0.009***	253 (67.8)	149 (24.0)	***< 0.001***
P-tau, pg/mL [mean (SD)]	16.5 (7.59)	15.4 (5.18)	17.8 (9.43)	***0.003***	21.6 (7.78)	11.5 (2.01)	***< 0.001***
sTREM2, pg/mL [mean (SD)]	7956 (2257)	8070 (2220)	7842 (2293)	*0.313*	8964 (2399)	7161 (1711)	***< 0.001***
Neurogranin, pg/mL [mean (SD)]	800 (331)	787 (296)	812 (362)	*0.452*	1047 (313)	587 (134)	***< 0.001***
𝛂- synuclein, pg/mL [mean (SD)]	234 (254)	235 (227)	232 (279)	*0.905*	298 (329)	184 (164)	***< 0.001***
S100B, pg/mL [mean (SD)]	1024 (236)	999 (209)	1049 (258)	***0.031***	1087 (250)	966 (206)	***< 0.001***
GFAP, pg/mL [mean (SD)]	7720 (2638)	7675 (2842)	7766 (2424)	*0.731*	8814 (2837)	6718 (2091)	***< 0.001***

(1) Other: Gypsy ethnic, latin american and not evaluated (2) Aβ42/Aβ40 median = 0.08057 (3) The cut-off for amyloid (A) positivity according to the Aβ42/Aβ40 ratio is 0.071. (4) P-tau median = 14.75 pg/mL. (5) The cut-off for tau (T) positivity according to P-tau is 24 pg/mL. LP: lumbar puncture. PH: Parental history. MMSE: mini-mental state examination score

The linear regression models showed associations between sTREM2 and synapse-related and astroglial biomarkers, summarized in Supplementary Table 3 and represented in Supplementary Fig. 2. As in the WRAP cohort, these associations were attenuated after adjusting for Aβ42 and p-tau levels, indicating partial dependence on AD pathology (Model 2). However, negative associations remained between sTREM2 and neurogranin (β = -0.21, *p* = 0.04), as well as in the subgroups with below-median p-tau (β = -0.37, *p* = 0.002) and T- (β = -0.21, *p* = 0.04). In contrast to WRAP cohort results, sTREM2 and ⍺-synuclein showed positive adjusted associations only in the below-median Aβ42/Aβ40 ratio and A + group (β = 0.15, *p* = 0.02 and β = 0.20, *p* = 0.007, respectively). Consistent with WRAP results, the ALFA + cohort showed a significant positive association between sTREM2 and S100B in the whole sample after adjusting for Aβ42 and p-tau (β = 0.28, *p* = 0.00002). This association was also significant in almost all subgroups, particularly in the A + group (β = 0.51, *p* = 0.000001). Furthermore, there was a significant interaction between S100B and Aβ42 levels in the whole cohort (*p* = 0.03). In contrast to the WRAP results, the ALFA + cohort demonstrated a positive, significant adjusted association between GFAP and sTREM2 in the whole sample (β = 0.24, *p* = 0.000002), which was also significant in most subgroups, except for T + and below-median Aβ42/Aβ40 ratio. Remaining interaction terms were tested using continuous values of Aβ42 and p-tau; none were statistically significant and are not shown.

Longitudinally, the linear mixed models revealed similar trends, with associations found only between synapse-related biomarkers and sTREM2, but not between glial activation biomarkers and sTREM2. They are summarized in Supplementary Table 4 and represented in Supplementary Fig. 3. Importantly, in contrast to the WRAP cohort results, the adjustment for Aβ42 and p-tau influenced the coefficients in Model 2. For neurogranin, we found an association between lower baseline neurogranin and a larger longitudinal increase in sTREM2 in Model 1, only in the above-median Aβ42/Aβ40 ratio group (β-coefficient for interaction with time = -0.02, *p* = 0.02) and the A- group (β-coefficient for interaction with time = -0.002, *p* = 0.008), suggesting an association in participants without evidence of amyloid pathology. However, these associations did not remain significant after adjusting for Aβ42 and p-tau (Model 2), suggesting confounding by AD pathology.

Similarly, we observed that lower baseline α-syn was associated with a larger longitudinal increase in sTREM2 over time in the above-median Aβ42/Aβ40 ratio group for both model 1 (β-coefficient for interaction with time = -0.01, *p* = 0.05) and model 2 (β-coefficient for interaction with time = -0.01, *p* = 0.05). As in the WRAP cohort, sTREM2 levels above the median were associated with a diminished longitudinal increase in neurogranin (β-coefficient for interaction with time = -0.01, *p* = 0.04), represented in Supplementary Fig. 4. In sum, while both cohorts demonstrated cross-sectional associations between sTREM2 and markers of synaptic and glial function, the longitudinal patterns were more robust and AD-independent in the WRAP cohort compared to ALFA+.

## Discussion

This study offers new insights into the interplay between synaptic dysfunction, TREM2-dependent microglial response, and astroglial activation, through a CSF-based biomarker approach applied to two independent longitudinal cohorts of cognitively normal, late-middle-aged individuals. Longitudinally, lower baseline levels of α-syn and neurogranin, and higher levels of S100B predicted a larger subsequent increase in sTREM2 in individuals with a biomarker profile non suggestive of an underlying AD pathological process. These findings suggest that early synaptic dysfunction may act as a trigger for TREM2-dependent microglial activation, regardless of AD pathology. Additionally, higher baseline levels of sTREM2 were associated with more stable neurogranin levels over time, further supporting the role of TREM2 as a modulator of synaptic function and potentially protective against synaptic dysregulation and cognitive decline throughout aging and early stages of neurodegenerative processes.

Cross-sectionally, we found an association between sTREM2 and α-syn, specifically in participants with neurodegeneration-related biomarker profiles (T + group in the WRAP cohort, and Aβ42/Aβ40 below median and A + groups in ALFA+). Interpreting CSF α-syn levels remains challenging due to its dual role in pathological processes, such as aggregation and neurodegeneration in synucleinopathies, and physiological processes, including synaptic function and axonal remodeling during normal aging [18, 27]. In neurodegenerative diseases, total CSF α-syn is often considered a marker of neurodegeneration rather than synaptic dysfunction [24, 39]. This is supported by previously reported significant correlations between ⍺-syn, p-tau and t-tau in CSF, in concordance with our results [25, 40]. However, in PD, most studies report reduced total CSF ⍺-syn levels during the early stages, likely reflecting initial synaptic dysfunction or early α-syn aggregation. In later stages, higher levels have been reported, which may signal overt neuronal injury [41]. The variations in α-syn levels clearly illustrate that it must be interpreted within its biological context. We interpret the observed cross-sectional relationship between sTREM2 and α-syn in participants with neurodegeneration-related biomarker profiles as reflective of a shared cross-sectional association with incipient neurodegeneration.

Regarding the cross-sectional relationship between sTREM2 and astroglial activation markers, we found a significant association between sTREM2 and S100B in the ALFA + cohort and a trend toward an association in the WRAP cohort. These associations remained robust after adjusting for AD-related biomarkers and were stronger in subgroups with a biochemical profile suggestive of early phases of AD pathology. This observation is consistent with previous studies that show stronger sTREM2–S100B correlations in asymptomatic participants with elevated p-tau/Aβ42 ratios [25] and across the symptomatic AD continuum [40, 42]. This suggests increased interaction between astroglial and microglial responses in early AD. Interestingly, we observed no cross-sectional association between GFAP and sTREM2 in the WRAP cohort. In contrast, both S100B and GFAP demonstrated significant cross-sectional associations with sTREM2 across subgroups in the ALFA + cohort.

Previous research has already described a relatively low correlation between S100B and other astroglial biomarkers [25], which suggests that S100B represents a distinct response specifically related to synaptic dysfunction rather than general astrocyte activation. Given its theoretical dual role—neurotrophic at nanomolar and pro-inflammatory at micromolar concentrations—S100B may reflect a neuroprotective astroglial response to early synaptic dysfunction in asymptomatic late-middle-aged individuals [43, 44]. This interpretation is reinforced by the weak correlation between S100B and GFAP, suggesting S100B secretion occurs independently from the pathological astrocytic hyperactivation indicated by increased GFAP. Differences between the WRAP and ALFA + cohorts could stem from their distinct participant characteristics, as the ALFA + cohort includes healthy volunteers enriched for AD risk factors, likely leading to earlier GFAP elevations as an initial astroglial response to pathology. In fact, the observed cross-sectional association between GFAP and sTREM2 in the ALFA + cohort probably reflects the early interplay between astroglial and microglial activation as AD-related changes begin. Overall, our findings indicate distinct astroglial response patterns involving S100B and GFAP across cohorts with varying AD risk profiles. This underscores a complex interplay between astroglial and microglial activation that may differentially reflect synaptic dysfunction and initial amyloid-related pathology.

Longitudinally, we observed that a biomarker profile suggestive of early synaptic dysfunction at baseline —characterized by lower levels of α-syn and neurogranin along with higher levels of S100B in CSF— was associated with a greater subsequent longitudinal increase of sTREM2 in CSF over time. These associations were independent of AD-related biomarker status in the WRAP cohort of cognitively healthy, late-middle-aged individuals. As discussed, interpreting CSF α-syn levels is challenging due to its involvement in different pathological and physiological processes [18, 27]. In participants of the WRAP cohort, who are mainly individuals without manifest amyloid deposition nor neurodegeneration, we interpret lower CSF α-syn levels as indicative of age-related synaptic dysfunction rather than incipient neurodegeneration. In contrast, in individuals with overt neurodegenerative processes or more evident AD pathology, α-syn levels may represent the underlying neurodegeneration, which our cross-sectional findings support. In the ALFA + cohort, lower levels of α-syn at baseline were associated to greater increases in sTREM2 specifically among participants with higher Aβ42/Aβ40 ratios. This highlights how synaptic dysfunction could be a booster of microglial activation within aging or non-AD neurodegeneration rather than within the AD continuum.

Similarly, lower neurogranin levels in individuals without biomarker evidence of AD likely reflect reduced postsynaptic activity. This interpretation is consistent with prior findings in PD, where neurogranin is reduced and correlates with cortical hypometabolism and cognitive deficits [45]. However, elevated CSF neurogranin has been reported even at presymptomatic stages in the AD continuum, likely reflecting neurodegeneration or neuronal hyperactivity rather than isolated synaptic dysfunction [17, 21, 22, 46–48]. The strong correlation between neurogranin and tau-related markers supports its role as a disease-stage specific injury marker of AD [22, 46]. Notably, increased network excitability—along with heightened seizure susceptibility—has been observed in early AD and may contribute to regional increases in synaptic density and elevated neurogranin levels [49], highlighting its dynamic, context-dependent interpretation. In the ALFA + cohort, lower baseline neurogranin was also associated with increased sTREM2 over time. However, only in subgroups without amyloid pathology and prior to adjustment for p-tau, suggesting that tau pathology may mask this association in a cohort with a high AD-risk profile.

Further supporting early synaptic dysfunction as an independent trigger for TREM2-dependent microglial activation, we found that individuals with higher S100B levels at baseline exhibited a greater longitudinal increase in sTREM2 in the WRAP cohort. Interestingly, baseline GFAP levels were not predictive of longitudinal changes in sTREM2 in the WRAP nor in the ALFA + cohort. The absence of longitudinal associations with GFAP suggests that generalized astroglial activation does not independently boost microglial responses over time. Instead, the selective longitudinal relationship between elevated S100B and subsequent sTREM2 increase reinforces our interpretation of S100B as a synapse-coupled astroglial signal that can prime microglia independently of GFAP-defined astrocytic activation during physiological aging and non-AD neurodegenerative processes. Mechanistically, astrocytic S100B may mark dendritic-spine stress that, in turn, triggers a TREM2-dependent pruning response, echoing the complement-mediated synapse-elimination pathway observed in recent mouse and human studies [50]. In the context of AD pathology, however, the strong effect of Aβ aggregation in boosting TREM2-dependent microglial response may override the subtler modulatory influence of synaptic dysfunction throughout aging and non-AD neurodegeneration processes.

And finally, we found that higher baseline CSF sTREM2 predicted a slower rise in neurogranin yet had no influence on α-syn or S100B trajectories. This pattern is congruent with recent findings that show TREM2-competent microglia identify phosphatidylserine-tagged, hyperactive spines and remove them via a complement pathway, thereby normalizing circuit activity both in disease models and clinical cohorts [50–55]. Instead, loss-of-function TREM2 variants in mice increase spine density, drive cortical hyperexcitability, and heighten seizure susceptibility [53]. This might be mirrored in electrophysiological phenotypes of patients with early AD who experience a higher incidence of subclinical and overt epileptic events [49]. Together, our findings and external evidence support a modulatory effect of TREM2-activated microglia on excessive synaptic activity: pruning superfluous spines, stabilizing neurogranin release, and reducing network hyperexcitability and seizure risk. The absence of longitudinal effects on the presynaptic marker α-syn or on astroglial S100B emphasizes that this microglial feedback loop is largely postsynaptic-specific.

This study has several limitations. First, the inclusion of only cognitively unimpaired individuals limits generalizability to symptomatic stages, though it enables the study of preclinical processes. Second, CSF biomarkers do not fully reflect regional synaptic or neuroinflammatory dynamics and the limited panel analyzed here may not comprehensively capture the complexity of these biological processes. Third, modest sample size and limited longitudinal data reduce statistical power and temporal resolution. Fourth, strong inter-biomarker correlations may obscure independent effects, despite the use of complementary sTREM2 assays. The use of cohort-specific Aβ and tau cut-offs may affect comparability across studies. Additionally, despite adjusting for p-tau, residual confounding cannot be excluded due to its collinearity with synaptic markers. Finally, due to the nature of this study, multiple comparison corrections were not performed, which may have led to overestimation of certain associations.

Despite these limitations, our study has several strengths. One key strength is the availability of longitudinal CSF biomarker data from cognitively normal individuals. This allows us to examine biomarker trajectories over time, providing valuable insights into the evolution of synaptic and glial markers in aging and the preclinical stages of neurodegeneration. Another strength is the validation of findings in a secondary cohort of cognitively normal individuals, enhancing the robustness of our results. Furthermore, we incorporated both pre-synaptic and post-synaptic biomarkers, as well as astroglial and microglial markers, thereby broadening the assessment of synaptic dysfunction and glial activity.

Together, our cross-sectional and longitudinal findings suggest that synaptic stress activates a TREM2-dependent microglial response across aging and in non-AD neuropathological contexts. Once Aβ aggregation begins along the AD-continuum, its potent effect on activating the TREM2 pathway may dominate, diminishing the relative contribution of synaptic dysfunction. Astroglial–microglial coupling also changes with AD biomarker status, emphasizing the dynamic and coordinated glial response to early neuropathological changes. Our findings support ongoing therapeutic strategies that target the synapse-to-glia axis, boosting TREM2 signaling or protecting synapses [29, 56], as potential early disease-modifying treatments for neurodegenerative diseases, and preventive interventions for cognitive decline through aging. The data highlights that the clinical benefit of modulating TREM2 may hinge on whether synaptic stress or established Aβ/tau pathology is the predominant biological driver.

Beyond therapeutic implications, our data reinforce the potential of a multimodal fluid biomarker panel—including markers of pre- and post-synaptic function (α-syn, neurogranin) and astroglial/microglial activation (s100b, GFAP, sTREM2)—to define distinct biochemical endophenotypes. These profiles may improve our ability to predict cognitive decline both in aging and AD, and to stratify individuals in early-phase clinical trials based on their synaptic state and glial reactivity. By capturing these upstream, modifiable processes before the onset of irreversible neuronal damage, such approaches hold promise for preventive strategies aimed at preserving cognitive health across the aging continuum.

## Supplementary Information

Below is the link to the electronic supplementary material.

Supplementary Material

## Data Availability

Data utilized in the WRAP cohort consist of sensitive, human research participant data and participants have not signed informed consent to have their data shared in public repositories for publications. Therefore, data deposition is unethical. Data are available upon request for authorized researchers who meet the criteria for access to confidential data. The data underlying the results presented in this study are available from [http://www.wai.wisc.edu/research/] (http://www.wai.wisc.edu/research/). Data from the ALFA+ cohort is also available upon request from [https://www.barcelonabeta.org/es/estudio-alfa/sobre-el-estudio-alfa] (https://www.barcelonabeta.org/es/estudio-alfa/sobre-el-estudio-alfa).

## Abbreviations

AD: Alzheimer’s disease
ALFA: ALzheimer and Families cohort
Aβ: Amyloid-β
CSF: Cerebrospinal fluid
DLB: Dementia with Lewy bodies
GFAP: Glial fibrillary acid protein
LP: Lumbar puncture
NTK: NeuroToolKit
PD: Parkinson’s disease
S100B: S100 calcium-binding protein B
sTREM2: Soluble cleavage product of the microglial protein TREM2
TREM2: Triggering receptor on myeloid cells 2
WRAP: Wisconsin Registry for Alzheimer’s Prevention
α-syn: Alpha-synuclein
