# Supplementary material for: Synaptic dysfunction and glial activation markers throughout aging and early neurodegeneration: a longitudinal CSF biomarker-based study
Source: Mol Neurodegener. 2025 Oct 17;20:109. doi: 10.1186/s13024-025-00901-5 (PMC12533437; doi:10.1186/s13024-025-00901-5)
Supplement: Supplementary file 1 — Supplementary Material [file 13024_2025_901_MOESM1_ESM.docx]

**Supplementary material**

Supplementary figure 1. Partial correlations adjusted by age between biomarkers at baseline


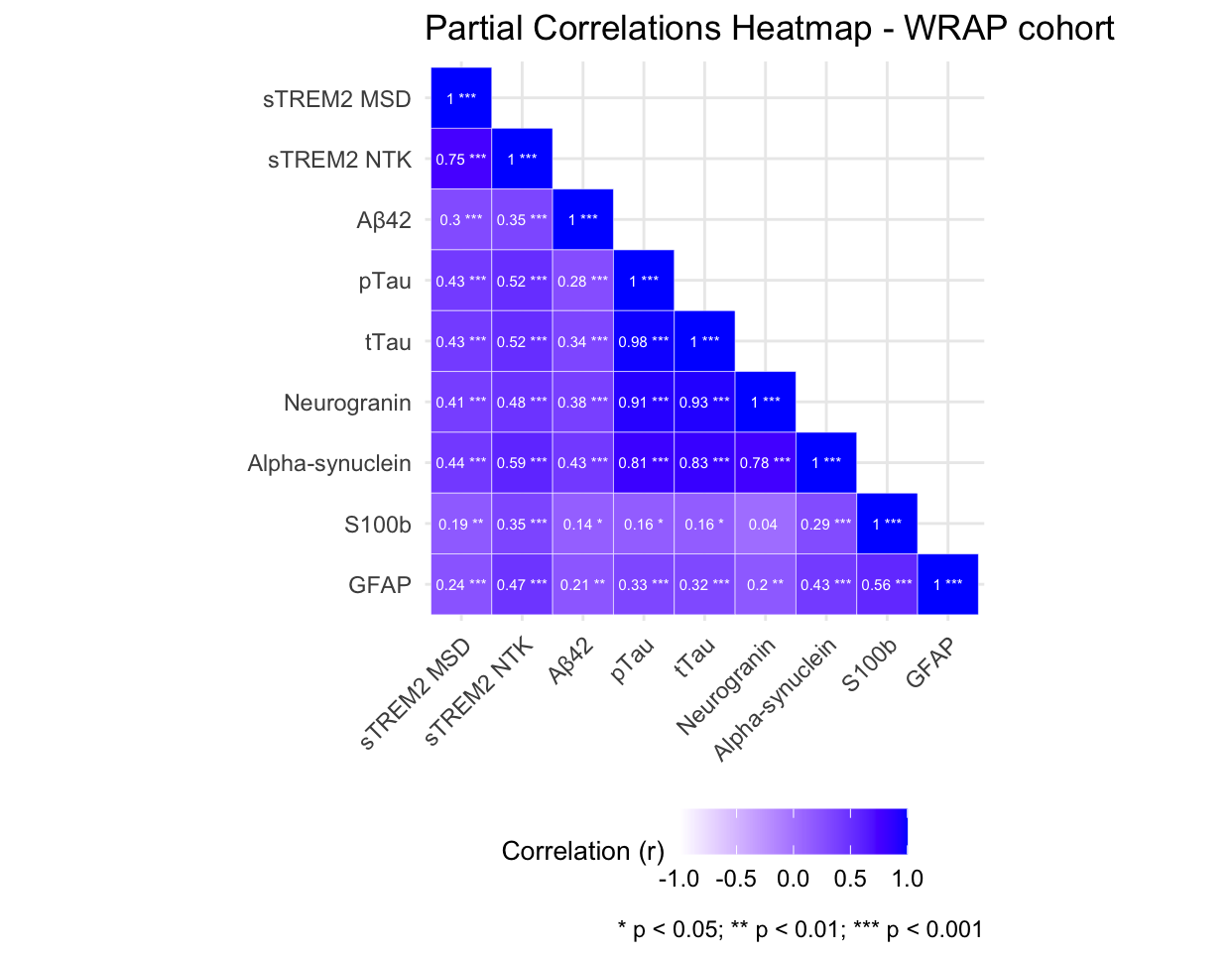


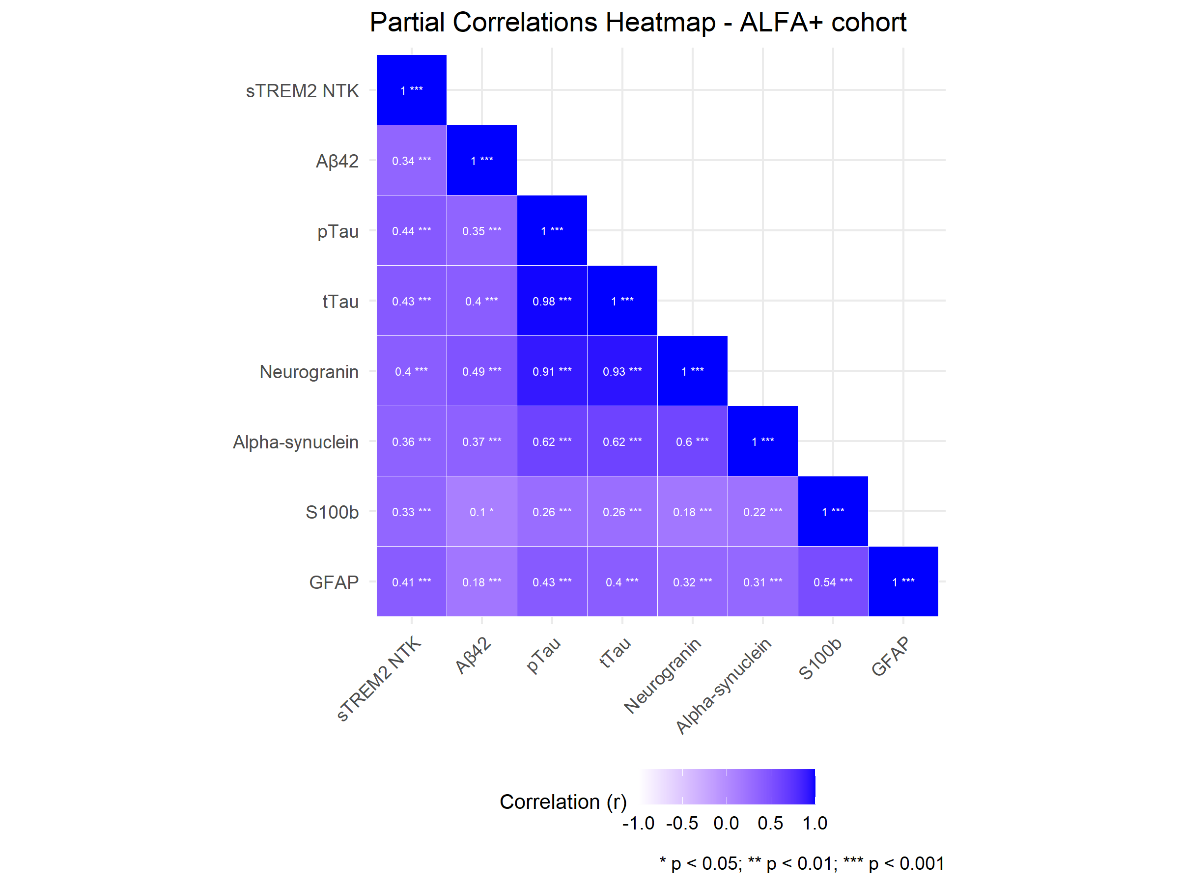


|  | Total sample | | < Aß42/Aß40 median | | > Aß42/Aß40 median | | A + | | A - | | < P-tau median | | > P-tau median | | T- | | T+ | | Apoe-e4 carrier | | Apoe-e4 non-carrier | |
| --- | --- | --- | --- | --- | --- | --- | --- | --- | --- | --- | --- | --- | --- | --- | --- | --- | --- | --- | --- | --- | --- | --- |
|  | n=239 | | n= 119 | | n=120 | | n= 46 | | n=193 | | n= 119 | | n=120 | | n=214 | | n=25 | | n=88 | | n=151 | |
|  | ß | *p-value* | ß | *p-value* | ß | *p-value* | ß | *p-value* | ß | *p-value* | ß | *p-value* | ß | *p-value* | ß | *p-value* | ß | *p-value* | ß | *p-value* | ß | *p-value* |
| Neurogranin | | | | | | | | | | | | | | | | | | | | | | |
| Model 1 | **0.37** | ***0.000*** | 0.38 | ***0.00*** | **0.35** | ***0.00*** | **0.40** | ***0.01*** | **0.39** | ***0.00*** | **0.25** | ***0.03*** | **0.27** | ***0.03*** | **0.39** | ***0.00*** | 0.49 | *0.29* | **0.39** | ***0.00*** | **0.36** | ***0.00*** |
| Model 2 | -0.04 | *0.78* | 0.13 | *0.48* | -0.37 | *0.08* | -0.05 | *0.88* | -0.05 | *0.73* | -0.04 | *0.81* | -0.06 | *0.77* | -0.07 | *0.61* | 0.32 | *0.59* | 0.10 | *0.59* | -0.16 | *0.39* |
| Alfa-synuclein | | | | | | | | | | | | | | | | | | | | | | |
| Model 1 | **0.37** | ***0.00*** | 0.37 | ***0.00*** | **0.39** | ***0.00*** | **0.48** | ***0.00*** | **0.37** | ***0.00*** | **0.30** | ***0.00*** | **0.29** | ***0.00*** | **0.37** | ***0.00*** | **0.79** | ***0.01*** | **0.40** | ***0.00*** | **0.36** | ***0.00*** |
| Model 2 | 0.16 | *0.09* | 0.15 | *0.25* | 0.20 | *0.14* | 0.37 | *0.08* | 0.12 | *0.26* | 0.11 | *0.42* | 0.19 | *0.141* | 0.11 | *0.25* | **0.83** | ***0.02*** | 0.20 | *0.19* | 0.14 | *0.24* |
| S100b | | | | | | | | | | | | | | | | | | | | | | |
| Model 1 | **0.28** | ***0.002*** | **0.41** | ***0.001*** | 0.15 | *0.26* | 0.29 | *0.21* | **0.28** | ***0.00*** | 0.11 | *0.33* | **0.32** | ***0.01*** | **0.22** | ***0.02*** | **0.74** | ***0.03*** | **0.28** | ***0.03*** | 0.24 | *0.06* |
| Model 2 | 0.16 | *0.06* | **0.26** | ***0.03*** | 0.06 | *0.61* | 0.23 | *0.27* | 0.15 | *0.10* | 0.05 | *0.64* | **0.28** | ***0.01*** | 0.10 | *0.23* | 0.70 | *0.07* | 0.18 | *0.12* | 0.11 | *0.37* |
| GFAP | | | | | | | | | | | | | | | | | | | | | | |
| Model 1 | **0.26** | ***0.0001*** | **0.21** | ***0.03*** | **0.31** | ***0.00*** | 0.27 | *0.17* | **0.26** | ***0.00*** | 0.08 | *0.36* | 0.12 | *0.18* | **0.23** | ***0.00*** | 0.40 | *0.22* | 0.20 | *0.05* | **0.31** | ***0.00*** |
| Model 2 | 0.09 | *0.15* | 0.04 | *0.67* | 0.16 | *0.07* | 0.14 | *0.47* | 0.10 | *0.16* | 0.11 | *0.28* | 0.08 | *0.36* | 0.09 | *0.18* | 0.32 | *0.42* | 0.07 | *0.46* | 0.13 | *0.14* |

**Supplementary table 1. WRAP cohort. Beta-coefficients and p-values for cross-sectional associations between synaptic biomarkers and cleaved soluble TREM2 (sTREM2)* in linear regression models**

| **Statistically significant (p<0.05)** |
| --- |
| *Model 1=adjusted by age and sex. Model 2=adjusted by age, sex,* Aβ42 *and PTAU* |
| Aβ42/Aβ40 *median =* 0.067. The cut-off for amyloid (A) positivity according to the Aβ42/Aβ40 ratio is < 0.046 |
| P-tau median = 15.94 pg/mL. The cut-off for tau (T) positivity according to P-tau is > 24.8 pg/mL |
| WRAP = Wisconsin Registry for Alzheimer prevention cohort |
| *Cleaved sTREM2 was measured by using an in-house MSD-based assay. |

**Supplementary table 2. WRAP cohort: Beta coefficients for the interaction between baseline biomarkers and time. Predicting longitudinal change in cleaved sTREM2***

|  | Total sample | | < Aß42/Aß40 median | | > Aß42/Aß40 median | | A + | | A - | | < P-tau median | | > P-tau median | | T- | | T+ | | Apoe-e4 carriers | | Apoe-e4 non-carriers | |
| --- | --- | --- | --- | --- | --- | --- | --- | --- | --- | --- | --- | --- | --- | --- | --- | --- | --- | --- | --- | --- | --- | --- |
|  | n=116 | | n= 61 | | n=55 | | n= 28 | | n=88 | | n= 53 | | n=63 | | n=105 | | n=11 | | n=46 | | n=71 | |
|  | ß | *p-value* | ß | *p-value* | ß | *p-value* | ß | *p-value* | ß | *p-value* | ß | *p-value* | ß | *p-value* | ß | *p-value* | ß | *p-value* | ß | *p-value* | ß | *p-value* |
| Neurogranin | | | | | | | | | | | | | | | | | | | | | | |
| Model 1 | **-0.041** | ***0.000*** | **-0.039** | ***0.002*** | **-0.045** | ***0.010*** | **-0.057** | ***0.004*** | **-0.038** | ***0.003*** | **-0.052** | ***0.011*** | **-0.052** | ***0.011*** | **-0.040** | ***0.001*** | -0.092 | *0.078* | **-0.038** | ***0.005*** | **-0.044** | ***0.005*** |
| Model 2 | **-0.041** | ***0.000*** | **-0.039** | ***0.002*** | **-0.046** | ***0.008*** | **-0.058** | ***0.003*** | **-0.038** | ***0.002*** | **-0.052** | ***0.011*** | **-0.052** | ***0.010*** | **-0.041** | ***0.001*** | -0.093 | *0.076* | **-0.039** | ***0.005*** | **-0.044** | ***0.005*** |
| Alfa-synuclein | | | | | | | | | | | | | | | | | | | | | | |
| Model 1 | **-0.026** | ***0.003*** | **-0.023** | ***0.049*** | **-0.031** | ***0.019*** | -0.035 | *0.066* | **-0.025** | ***0.012*** | -0.028 | *0.119* | -0.024 | *0.100* | **-0.025** | ***0.010*** | -0.018 | *0.688* | -0.021 | *0.089* | **-0.030** | ***0.015*** |
| Model 2 | **-0.026** | ***0.002*** | -0.022 | *0.051* | **-0.031** | ***0.019*** | -0.035 | *0.066* | **-0.026** | ***0.011*** | -0.028 | *0.117* | -0.024 | *0.098* | **-0.025** | ***0.009*** | -0.018 | *0.689* | -0.021 | *0.083* | **-0.030** | ***0.015*** |
| S100b | | | | | | | | | | | | | | | | | | | | | | |
| Model 1 | 0.016 | *0.317* | 0.014 | *0.419* | 0.019 | *0.520* | 0.052 | *0.052* | 0.001 | *0.976* | 0.013 | *0.629* | 0.042 | *0.050* | 0.017 | *0.318* | 0.017 | *0.651* | 0.008 | *0.697* | 0.029 | *0.268* |
| Model 2 | 0.015 | *0.338* | 0.014 | *0.422* | 0.016 | *0.580* | **0.052** | ***0.050*** | -0.001 | *0.958* | 0.012 | *0.638* | **0.042** | ***0.049*** | 0.015 | *0.366* | 0.017 | *0.637* | 0.007 | *0.704* | 0.028 | *0.286* |
| GFAP | | | | | | | | | | | | | | | | | | | | | | |
| Model 1 | -0.003 | *0.770* | -0.002 | *0.858* | -0.005 | *0.766* | 0.045 | *0.046* | -0.015 | *0.243* | -0.002 | *0.932* | 0.016 | *0.300* | -0.002 | *0.863* | 0.037 | *0.240* | -0.003 | *0.832* | -0.003 | *0.841* |
| Model 2 | -0.004 | *0.731* | -0.002 | *0.871* | -0.007 | *0.682* | 0.046 | *0.044* | -0.015 | *0.220* | -0.002 | *0.904* | 0.016 | *0.302* | -0.003 | *0.802* | 0.037 | *0.232* | -0.004 | *0.802* | -0.003 | *0.831* |

| **Statistically significant (p<0.05)** |
| --- |
| *Model 1=adjusted by age and sex. Model 2=adjusted by age, sex,* Aβ42 *and p-tau* |
| **Cleaved sTREM2 was measured by using an in-house MSD-based assay.* |

**Supplementary table 3. ALFA+ Cohort. Beta-coefficients and p-values for cross-sectional associations between synaptic biomarkers and soluble TREM2 (sTREM2) in linear regression models**

|  | Total sample | | < Aß42/Aß40 median | | > Aß42/Aß40 median | | A + | | A - | | < P-tau median | | > P-tau median | | T- | | T+ | | Apoe-e4 carriers | | Apoe-e4 non-carriers | |
| --- | --- | --- | --- | --- | --- | --- | --- | --- | --- | --- | --- | --- | --- | --- | --- | --- | --- | --- | --- | --- | --- | --- |
|  | n=239 | | n= 119 | | n=120 | | n= 46 | | n=193 | | n= 119 | | n=120 | | n=214 | | n=25 | | n=46 | | n=71 | |
|  | ß | *p-value* | ß | *p-value* | ß | *p-value* | ß | *p-value* | ß | *p-value* | ß | *p-value* | ß | *p-value* | ß | *p-value* | ß | *p-value* | ß | *p-value* | ß | *p-value* |
| Neurogranin | | | | | | | | | | | | | | | | | | | | | | |
| Model 1 | **0.31** | ***0.00*** | **0.27** | ***0.00*** | **0.36** | ***0.00*** | **0.30** | ***0.00*** | **0.34** | ***0.00*** | **0.20** | ***0.01*** | **0.31** | ***0.00*** | **0.28** | **0.00** | 0.01 | 0.95 | **0.25** | **0.00** | **0.36** | **0.00** |
| Model 2 | **-0.21** | ***0.04*** | -0.13 | *0.43* | -0.23 | *0.06* | -0.26 | *0.16* | -0.14 | *0.25* | **-0.37** | ***0.00*** | -0.03 | *0.85* | **-0.22** | **0.04** | -0.20 | 0.57 | -0.05 | 0.76 | **-0.32** | **0.01** |
| Alfa-synuclein | | | | | | | | | | | | | | | | | | | | | | |
| Model 1 | **0.21** | ***0.00*** | **0.26** | ***0.00*** | **0.16** | ***0.00*** | **0.30** | ***0.00*** | **0.17** | ***0.00*** | **0.12** | ***0.00*** | **0.19** | ***0.00*** | **0.18** | ***0.00*** | 0.11 | *0.19* | **0.15** | ***0.00*** | ***0.25*** | ***0.00*** |
| Model 2 | 0.07 | *0.06* | **0.15** | ***0.02*** | 0.02 | *0.53* | **0.20** | ***0.01*** | 0.03 | *0.45* | 0.04 | *0.28* | 0.10 | *0.11* | 0.06 | *0.15* | 0.15 | *0.15* | *0.06* | *0.34* | ***0.08*** | ***0.05*** |
| S100b | | | | | | | | | | | | | | | | | | | | | | |
| Model 1 | **0.44** | ***0.00*** | **0.42** | ***0.00*** | **0.49** | ***0.00*** | **0.59** | ***0.00*** | **0.34** | ***0.00*** | **0.33** | ***0.00*** | **0.33** | ***0.00*** | **0.39** | ***0.00*** | 0.27 | *0.15* | ***0.35*** | ***0.00*** | ***0.63*** | ***0.00*** |
| Model 2 | **0.28** | ***0.00*** | **0.30** | ***0.00*** | **0.28** | ***0.00*** | **0.51** | ***0.00*** | 0.13 | *0.11* | **0.25** | ***0.00*** | **0.29** | ***0.00*** | **0.28** | ***0.00*** | 0.26 | *0.16* | ***0.26*** | ***0.01*** | ***0.34*** | ***0.00*** |
| GFAP | | | | | | | | | | | | | | | | | | | | | | |
| Model 1 | **0.40** | **0.00** | **0.34** | **0.00** | **0.44** | **0.00** | **0.44** | **0.00** | **0.27** | **0.01** | **0.30** | **0.00** | **0.30** | **0.00** | **0.36** | **0.00** | 0.11 | 0.51 | **0.37** | **0.00** | **0.26** | **0.00** |
| Model 2 | **0.24** | **0.00** | 0.16 | 0.07 | **0.29** | **0.00** | **0.38** | **0.00** | **0.22** | **0.00** | **0.23** | **0.00** | **0.26** | **0.00** | **0.25** | **0.00** | 0.17 | 0.33 | **0.43** | **0.00** | **0.23** | **0.00** |

| **Statistically significant (p<0.05)** |
| --- |
| *Model 1=adjusted by age and sex. Model 2=adjusted by age, sex,* Aβ42 *and PTAU* |
| Aβ42/Aβ40 *median =* 0.08057. The cut-off for amyloid (A) positivity according to the Aβ42/Aβ40 ratio is < 0.071. |
| P-tau median = 14.75 pg/mL. The cut-off for tau (T) positivity according to P-tau is > 24 pg/mL |
| ALFA = Alzheimer’s and Families cohort |

**Supplementary figure 2**. Cross-sectional associations between biomarkers and sTREM2 NTK in the ALFA+ cohort.

**Coefficients and confidence intervals for Neurogranin**

**Coefficients and confidence intervals for Alpha-synuclein**


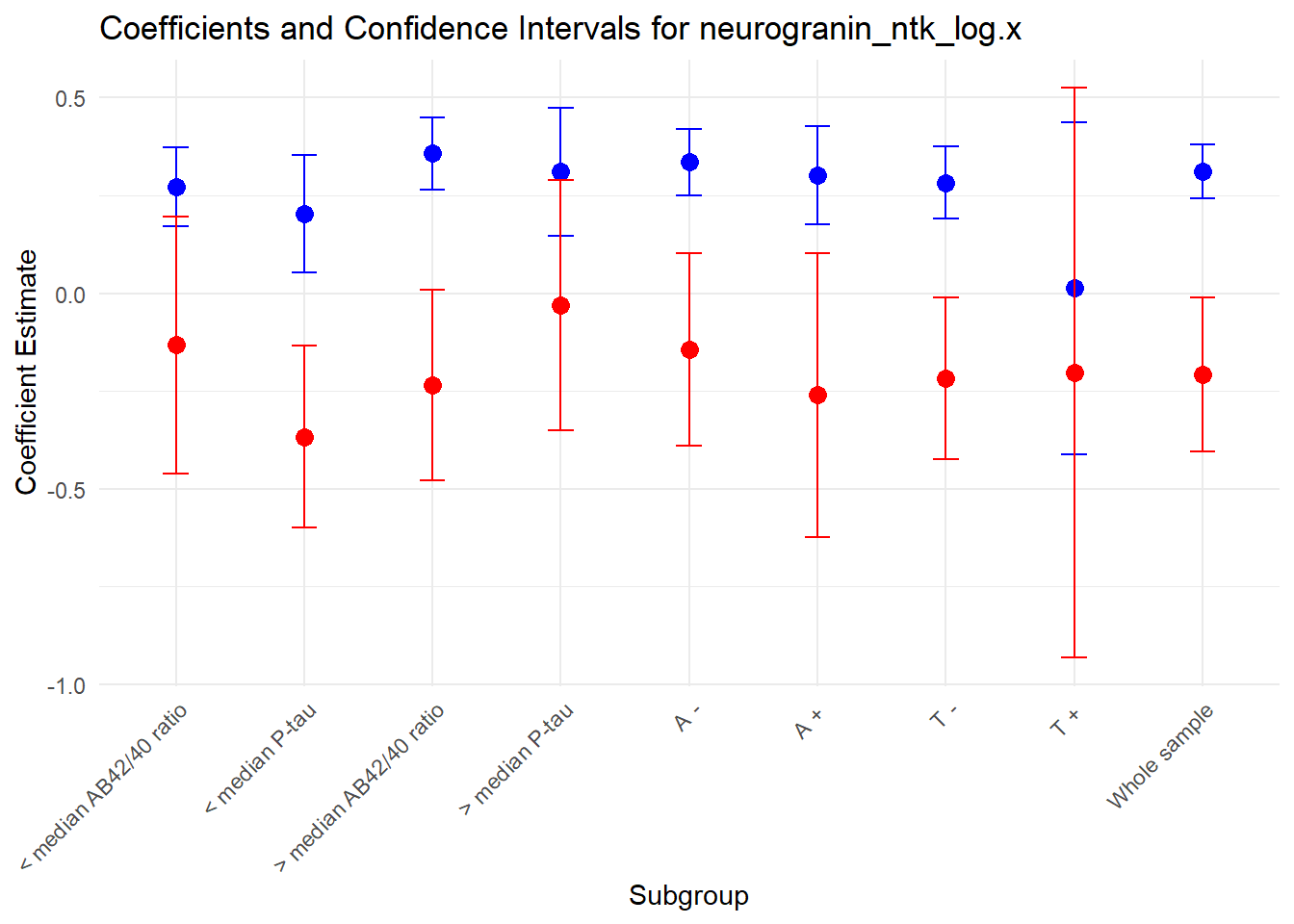

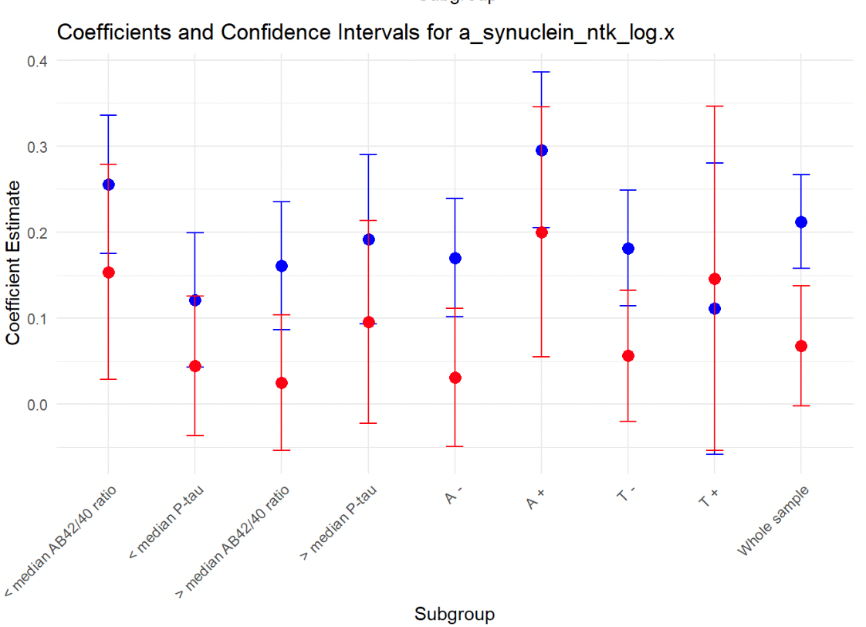


**Coefficients and confidence intervals for S100B**

**Coefficients and confidence intervals for GFAP**


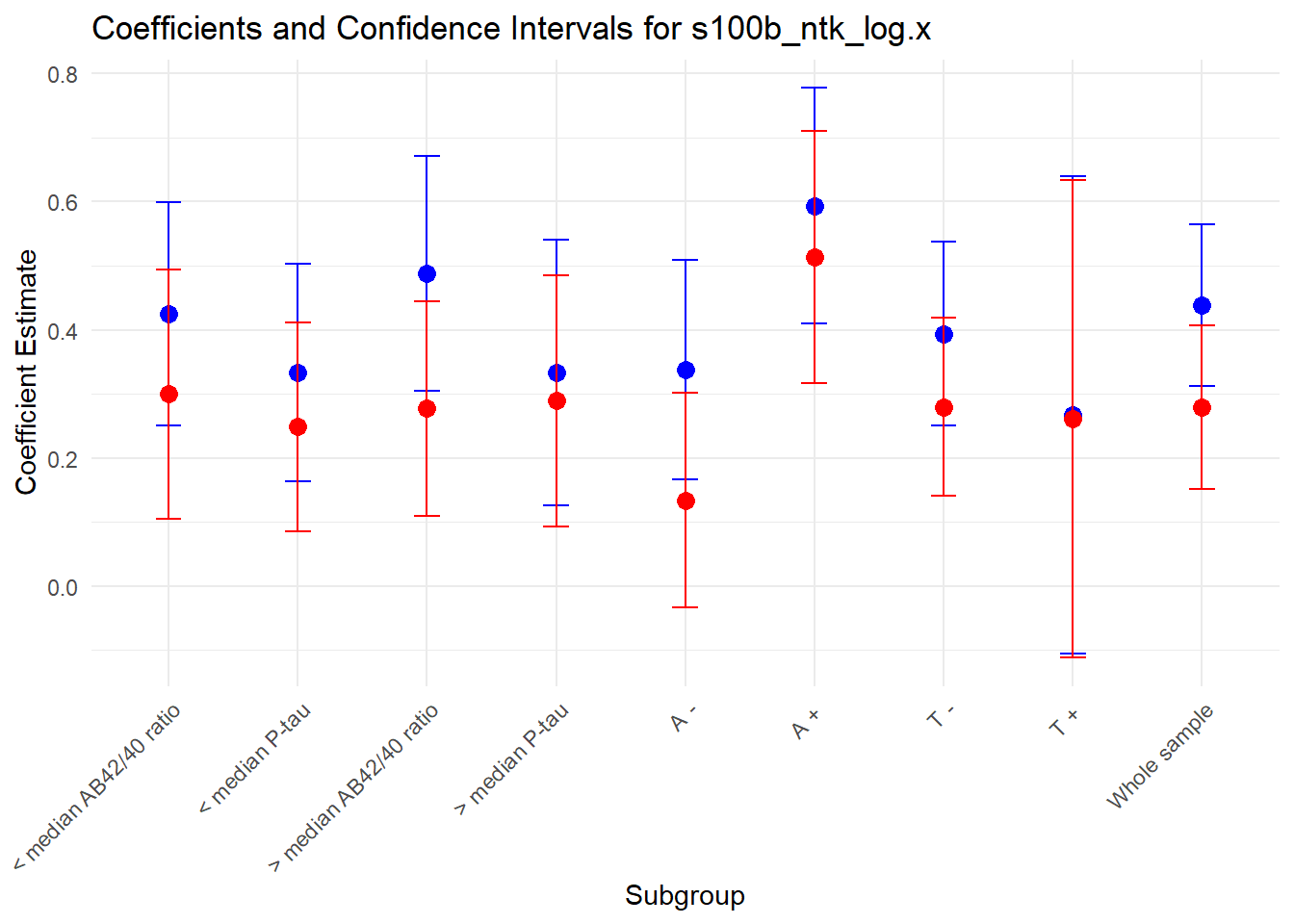

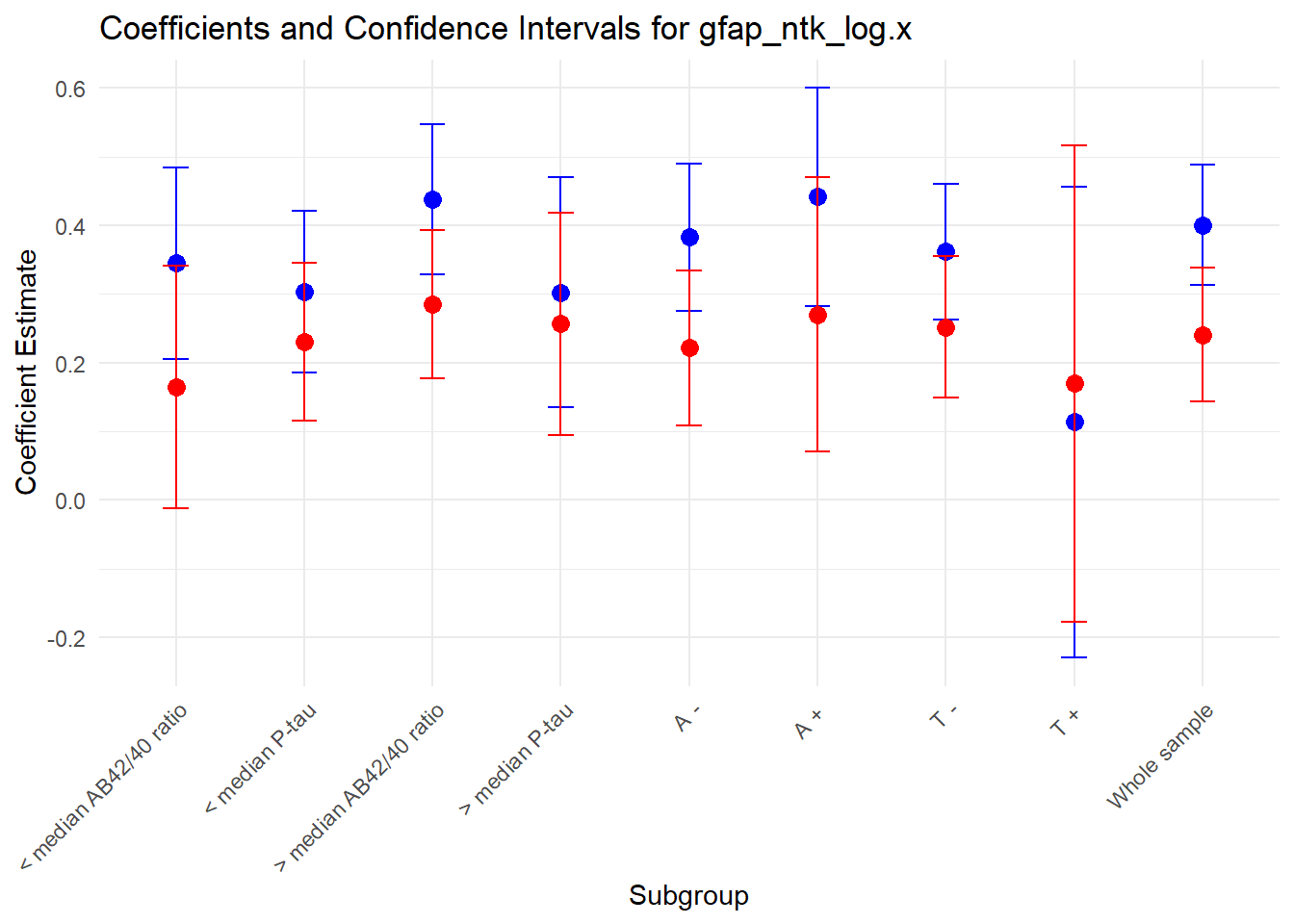


Linear regression models with each biomarker as the independent variable predicting sTREM2 levels. Model 1 (blue) is adjusted for age and sex. Model 1 (red) is adjusted for age, sex, Aβ42, and p-tau. Results are shown in the whole sample and stratified by subgroups according to medians (Aβ42/Aβ40 median = 0.067; p-tau median = 15.94 pg/mL), and cut-offs for amyloid (A+) positivity (Aβ42/Aβ40 < 0.046) and tau (T+) positivity (p-tau > 24.8 pg/mL). Error bars represent 95% confidence intervals.

**Supplementary table 4. ALFA+ cohort: Beta coefficients for the interaction between baseline biomarkers and time. Predicting longitudinal change in sTREM2**

|  | Total sample | | < Aß42/Aß40 median | | > Aß42/Aß40 median | | A + | | A - | | < P-tau median | | > P-tau median | | T- | | T+ | | Apoe-e4 carriers | | Apoe-e4 non-carriers | |
| --- | --- | --- | --- | --- | --- | --- | --- | --- | --- | --- | --- | --- | --- | --- | --- | --- | --- | --- | --- | --- | --- | --- |
|  | n=277 | | n= 135 | | n=142 | | n= 92 | | n=185 | | n= 115 | | n=129 | | n=212 | | n=32 | | n=152 | | n=125 | |
|  | ß | *p-value* | ß | *p-value* | ß | *p-value* | ß | *p-value* | ß | *p-value* | ß | *p-value* | ß | *p-value* | ß | *p-value* | ß | *p-value* | ß | *p-value* | ß | *p-value* |
| Neurogranin | | | | | | | | | | | | | | | | | | | | | | |
| Model 1 | -0.006 | *0.222* | 0.001 | *0.878* | **-0.015** | ***0.018*** | 0.007 | *0.476* | **-0.016** | ***0.008*** | -0.012 | *0.280* | 0.006 | *0.494* | -0.005 | *0.397* | 0.021 | *0.443* | -0.008 | *0.227* | -0.004 | *0.615* |
| Model 2 | -0.002 | *0.744* | 0.007 | *0.339* | -0.011 | *0.105* | -0.001 | *0.875* | -0.005 | *0.363* | -0.012 | *0.287* | 0.007 | *0.491* | -0.005 | *0.398* | 0.022 | *0.436* | -0.005 | *0.466* | 0.003 | *0.656* |
| Alfa-synuclein | | | | | | | | | | | | | | | | | | | | | | |
| Model 1 | -0.007 | *0.091* | -0.004 | *0.596* | **-0.011** | ***0.053*** | 0.002 | *0.776* | **-0.013** | ***0.010*** | -0.004 | *0.466* | 0.001 | *0.865* | -0.007 | *0.182* | 0.011 | *0.313* | **-0.013** | ***0.042*** | -0.003 | *0.619* |
| Model 2 | -0.002 | *0.630* | 0.007 | *0.240* | **-0.011** | ***0.048*** | 0.004 | *0.559* | -0.006 | *0.191* | -0.004 | *0.483* | 0.001 | *0.868* | -0.006 | *0.190* | 0.011 | *0.310* | -0.005 | *0.466* | 0.000 | *0.966* |
| S100b | | | | | | | | | | | | | | | | | | | | | | |
| Model 1 | 0.008 | *0.403* | 0.013 | *0.360* | -0.001 | *0.948* | 0.020 | *0.236* | 0.000 | *0.976* | 0.023 | *0.090* | -0.001 | *0.950* | 0.006 | *0.518* | 0.016 | *0.519* | 0.012 | *0.280* | 0.000 | *0.997* |
| Model 2 | 0.007 | *0.376* | 0.010 | *0.415* | 0.003 | *0.793* | 0.018 | *0.204* | -0.001 | *0.942* | 0.023 | *0.091* | -0.001 | *0.936* | 0.006 | *0.523* | 0.016 | *0.532* | 0.010 | *0.397* | 0.003 | *0.797* |
| GFAP | | | | | | | | | | | | | | | | | | | | | | |
| Model 1 | -0.001 | *0.832* | 0.000 | *0.961* | -0.002 | *0.773* | 0.003 | *0.788* | -0.005 | *0.490* | 0.005 | *0.563* | -0.003 | *0.745* | -0.002 | *0.734* | 0.013 | *0.488* | -0.005 | *0.595* | -0.002 | *0.831* |
| Model 2 | 0.000 | *0.978* | 0.001 | *0.921* | -0.001 | *0.885* | -0.002 | *0.827* | -0.001 | *0.891* | 0.005 | *0.558* | -0.003 | *0.745* | -0.002 | *0.736* | 0.013 | *0.493* | 0.001 | *0.895* | 0.002 | *0.820* |

| **Statistically significant (p<0.05)** |
| --- |
| *Model 1=adjusted by age and sex. Model 2=adjusted by age, sex,* Aβ42 *and p-tau* |

**Supplementary figure 3**. Longitudinal associations between baseline biomarkers and sTREM2 NTK in the ALFA cohort.

**Alpha-synuclein**

**Neurogranin**

**Neurogranin**


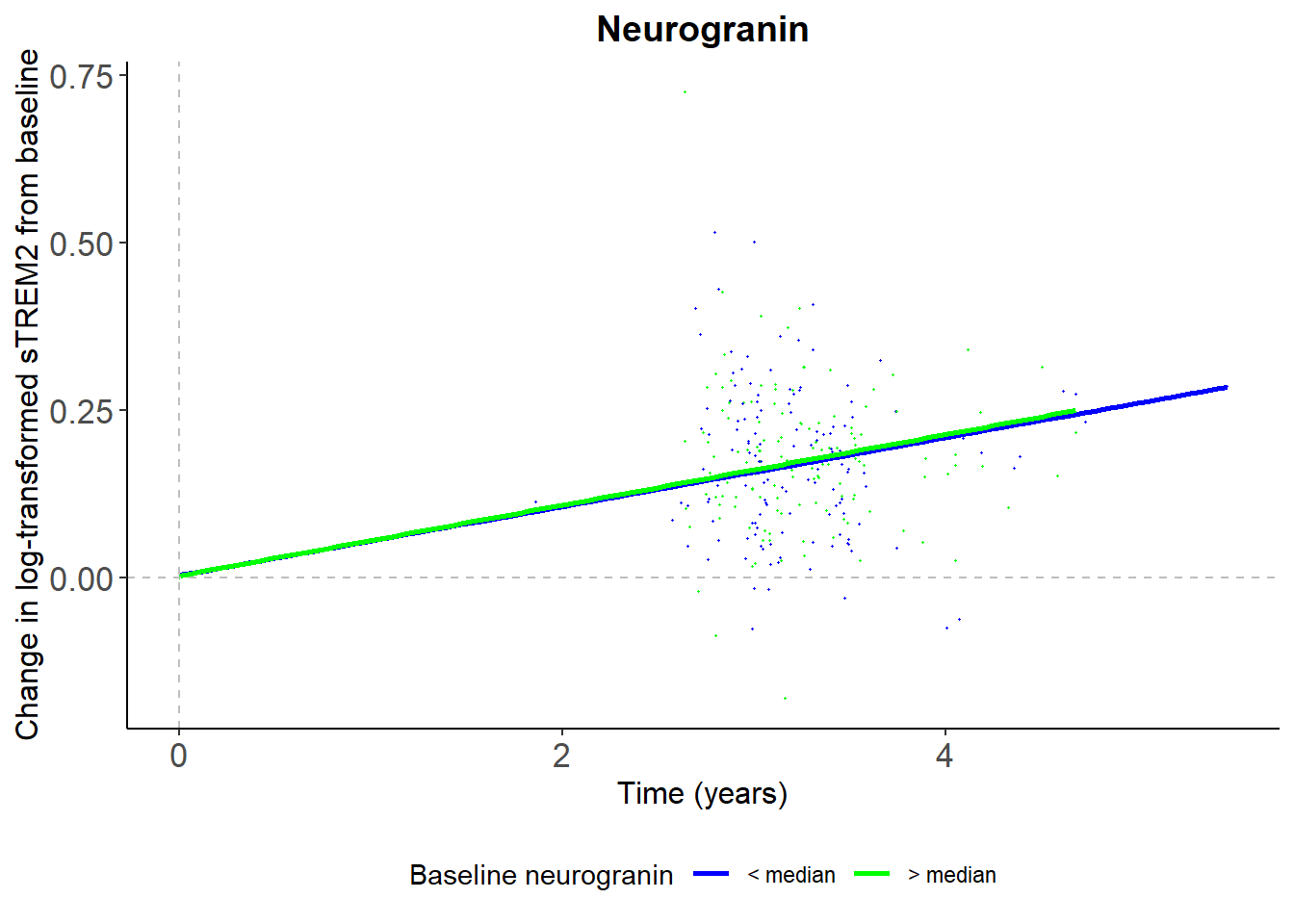

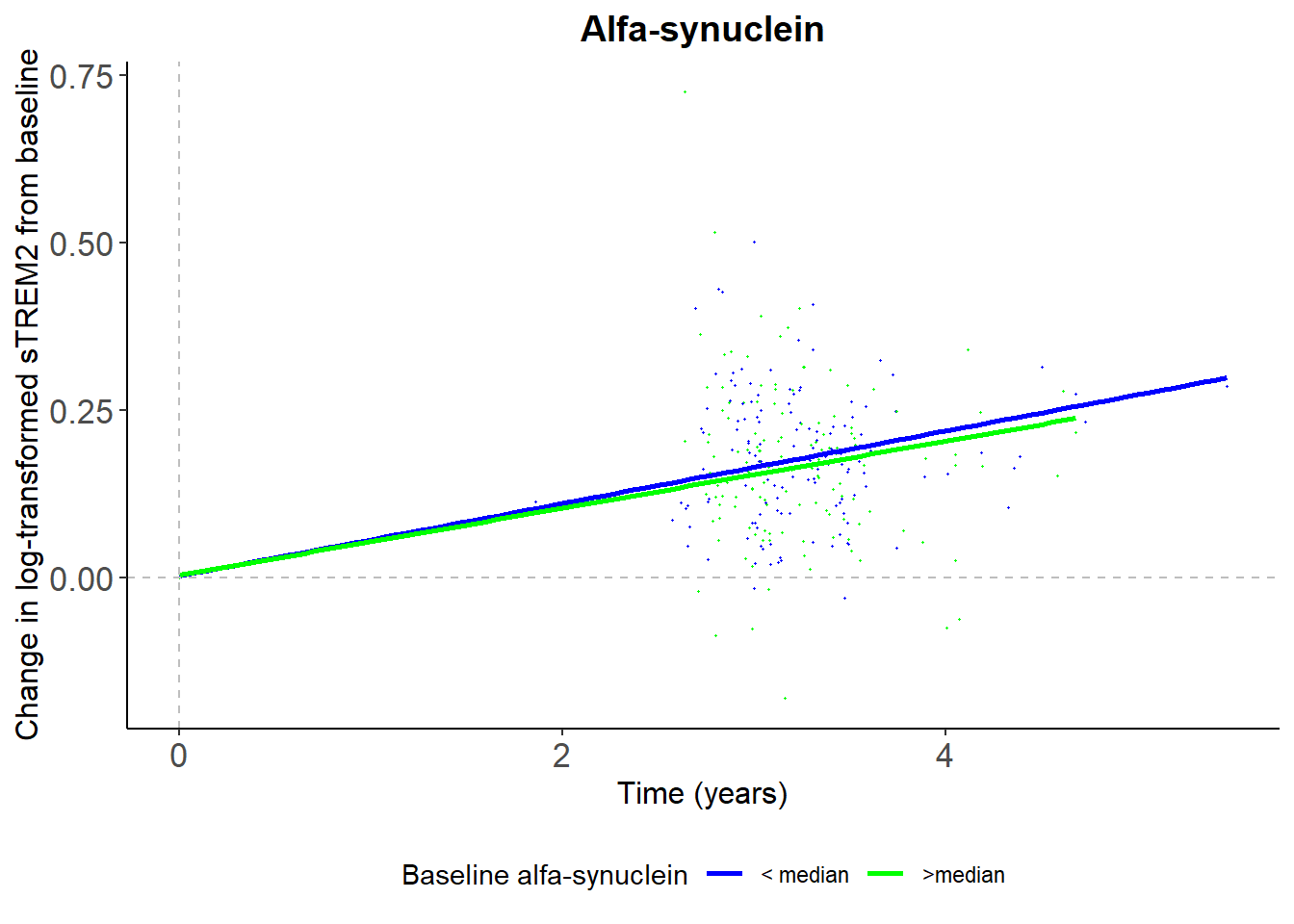


**GFAP**

**S100B**


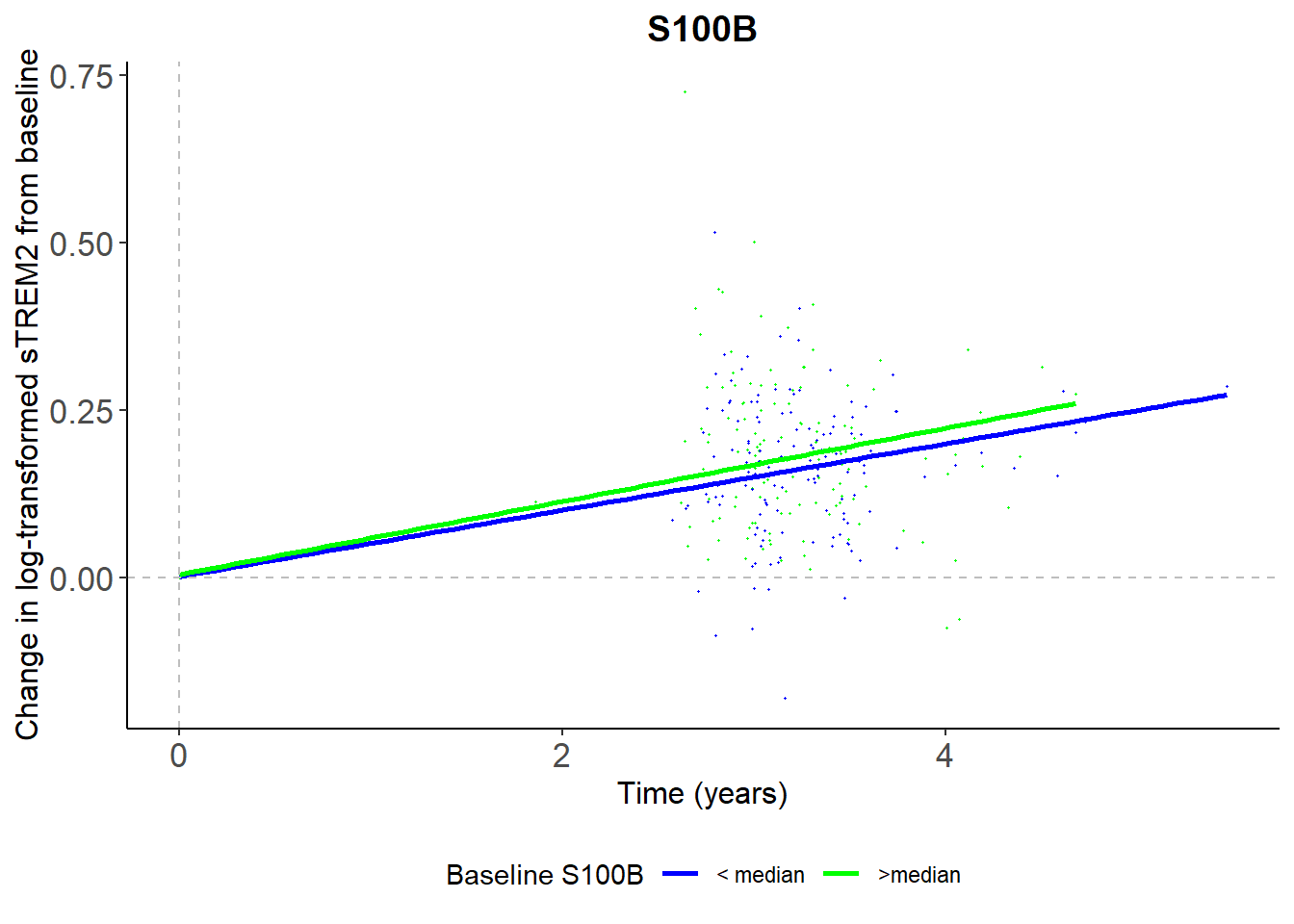

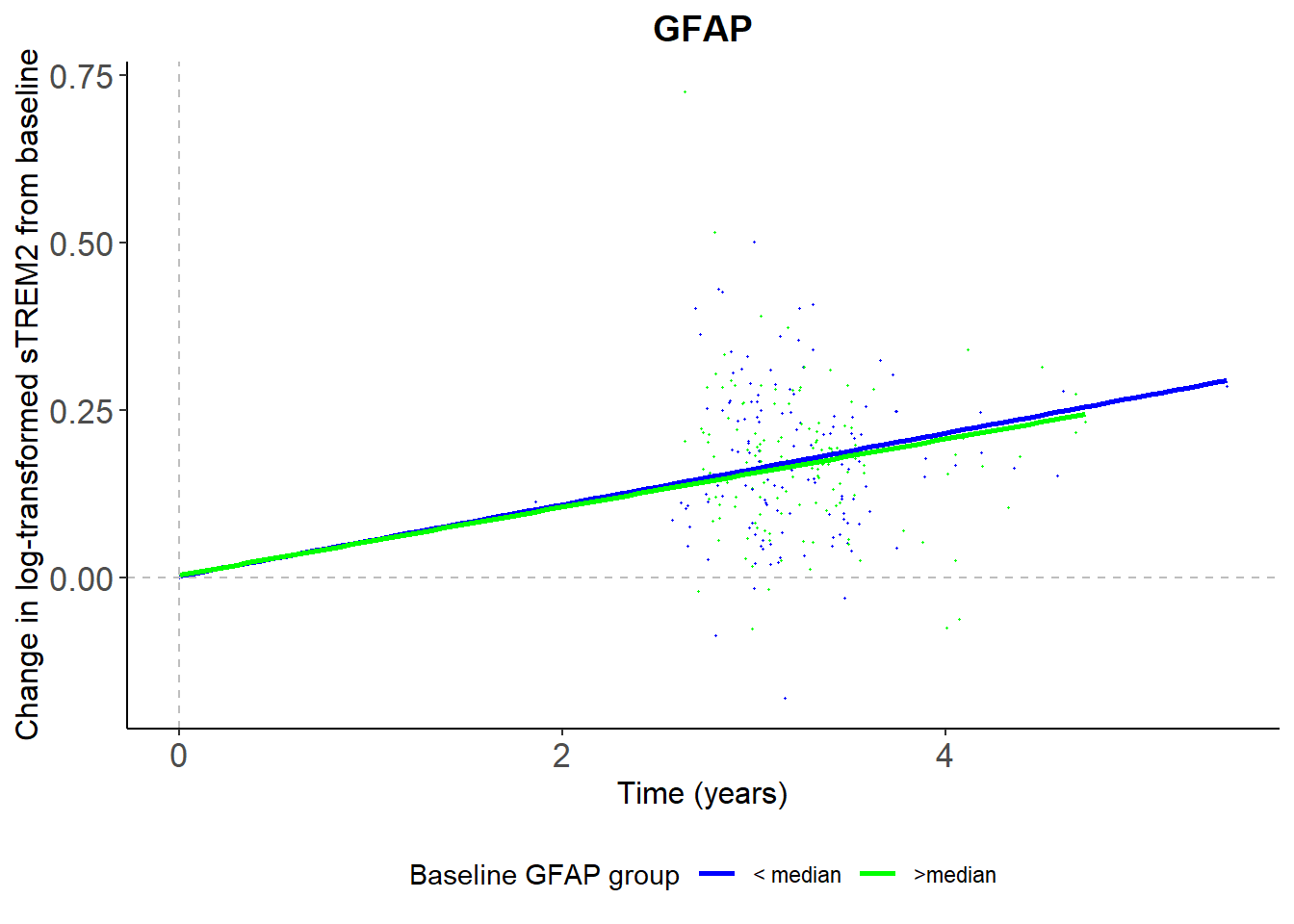


Figures show the change from baseline levels in log-transformed values across time for sTREM2, according to baseline levels of each biomarker. The dotted line represents the intercept (0). Linear mixed models that represent these plotted associations are adjusted by age, gender, AB42 and p-tau. *Neurogranin*: Beta-coefficient for interaction with time = -0.002, p= 0.74 (continuous values), Beta-coefficient for interaction with time = -0.005, p= 0.19 (comparing >median vs <median). *Alpha-synuclein*: Beta-coefficient for interaction with time = -0.002, p= 0.63 (continuous values), Beta-coefficient for interaction with time = -0.002, p= 0.57 (comparing >median vs <median). *S100b*: Beta-coefficient for interaction with time = 0.007, p= 0.38 (continuous values), Beta-coefficient for interaction with time = 0.004, p= 0.28 (comparing >median vs <median). *GFAP*: Beta-coefficient for interaction with time = -0.0001, p= 0.98 (continuous values). Beta-coefficient for interaction with time = -0.0001, p= 0.97 (comparing >median vs <median).

**Supplementary figure 4**. Longitudinal associations between baseline sTREM2 and biomarkers in the ALFA cohort.

**Alpha-synuclein trajectory according to baseline sTREM2**


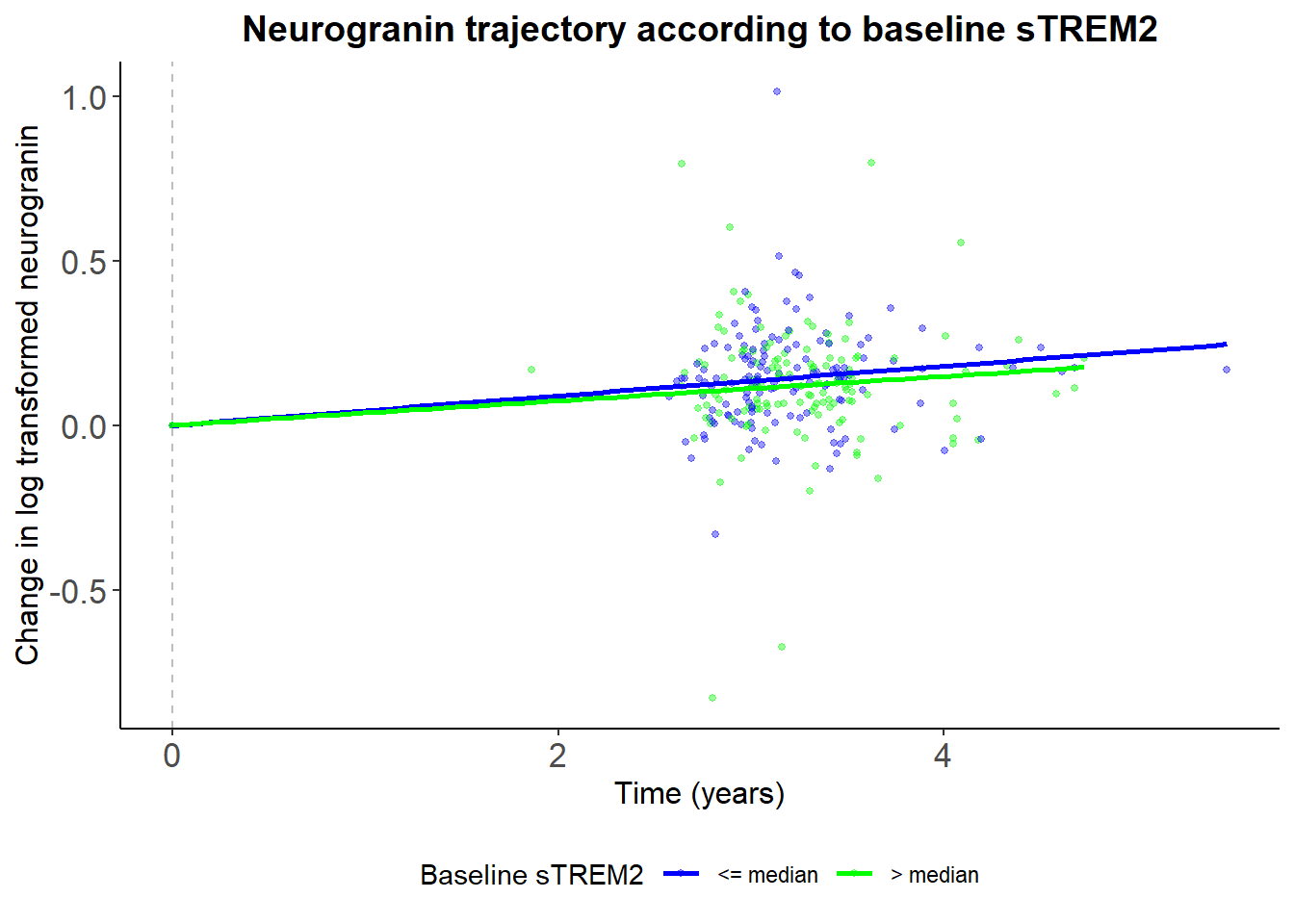

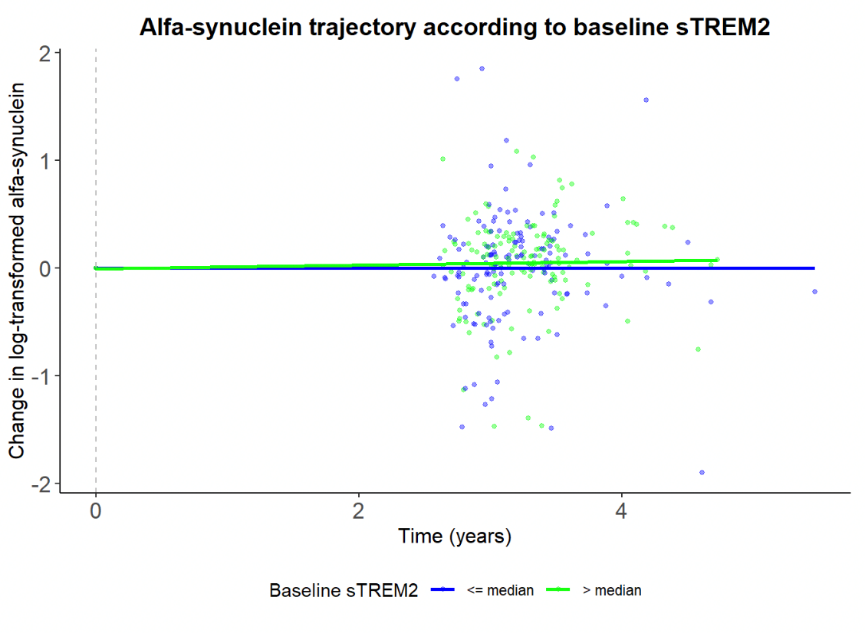


**S100B trajectory according to baseline sTREM2**

**Neurogranin trajectory according to baseline sTREM2**


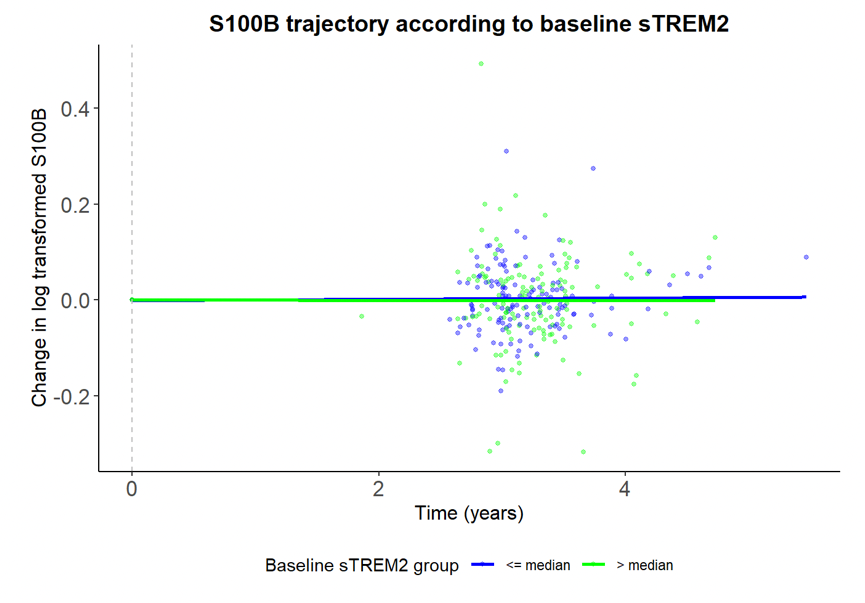

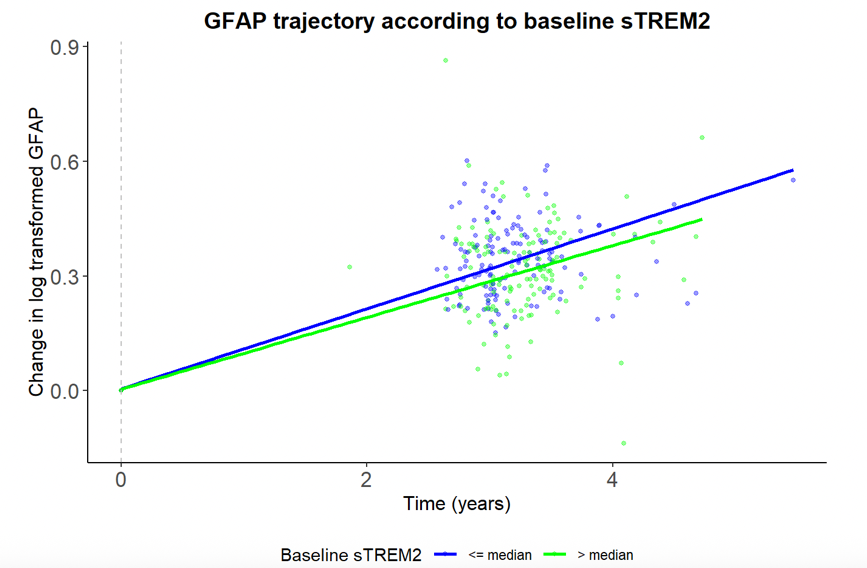


**GFAP trajectory according to baseline sTREM2**

Figures show the change from baseline levels in log-transformed values across time for each biomarker, according to baseline sTREM2 levels. The dotted line represents the intercept (0). Linear mixed models that represent these plotted associations are adjusted by age, gender, AB42 and p-tau. *Neurogranin*: "Beta-coefficient for interaction with time = -0.006, p= 0.58 (continuous values). Beta-coefficient for interaction with time = -0.01, p= 0.04 (comparing >median vs <median). *⍺-syn*: Beta-coefficient for interaction with time = -0.001, p= 0.97 (continuous values). = 0.005, p= 0.76 (comparing >median vs <median). *S100b*: Beta-coefficient for interaction with time = -0.004, p= 0.47 (continuous values). Beta-coefficient for interaction with time = -0.001, p= 0.65 (comparing >median vs <median). *GFAP*: Beta-coefficient for interaction with time = -0.009, p= 0.19 (continuous values). Beta-coefficient for interaction with time = -0.008, p= 0.03 (comparing >median vs <median).
